# Redox Self‐Equilibration in Molecular Vanadium Oxide Mixtures Enables Multi‐Electron Storage

**DOI:** 10.1002/anie.202418864

**Published:** 2024-11-16

**Authors:** Moritz Remmers, Boris Mashtakov, Stefan Repp, Alexandra Stefanie Jessica Rein, Ke Wang, Montaha Anjass, Zhengfan Chen, Luca M. Carrella, Eva Rentschler, Carsten Streb

**Affiliations:** ^1^ Department of Chemistry Johannes Gutenberg University Mainz Duesbergweg 10–14 55128 Mainz Germany; ^2^ Institute of Inorganic Chemistry I Ulm University Albert-Einstein-Allee 11 89081 Ulm Germany; ^3^ Department of Chemistry University of Sharjah P. O. Box 27272 500001 Sharjah United Arab Emirates

**Keywords:** Polyoxometalate, Polyoxovanadate, Self-Assembly, Vanadium Oxide, Electrochemistry

## Abstract

Polyoxometalates (POMs) are ideal components for reversible multi‐electron storage in energy technologies. To‐date, most redox‐applications employ only single, individual POM species, which limits the number of electrons that can be stored within a given potential window. Here, we report that spontaneous redox self‐equilibration during cluster synthesis leads to the formation of two structurally related polyoxovanadates which subsequently aggregate into co‐crystals. This results in systems with significantly increased redox reactivity. The mixed POM system was formed by non‐aqueous self‐assembly of a vanadate precursor in the presence of Mg^2+^, resulting in two mixed‐valent (V^IV/V^) species, [(MgOH)V_13_O_33_Cl]^4−^ (=**{MgV_13_}**) and the di‐vanadium‐functionalized species [V_14_O_34_Cl]^4−^ (=**{V_14_}**), which co‐crystallize in a 1 : 1 molar stoichiometry. Experimental data indicate that in the native state, **{MgV_13_}** is reduced by three electrons, and **{V_14_}** is reduced by five electrons. Electrochemical studies in solution show, that the system can reversibly undergo up to fourteen redox transitions (tentatively assigned to twelve 1‐electron processes and two 2‐electron processes) in the potential range between −2.15 V to +1.35 V (vs Fc^+^/Fc). The study demonstrates how highly redox‐active, well‐defined molecular mixtures of mixed‐valent molecular metal oxides can be accessed by redox‐equilibration during synthesis, opening new avenues for molecular energy storage.

## Graphical abstract

Rare co‐crystals of two mixed‐valent polyoxovanadate clusters are formed by a redox‐equilibration self‐assembly synthesis. The two‐cluster system shows remarkable redox‐activity with up to 16 redox‐transitions in a potential range of ~3.5 V.

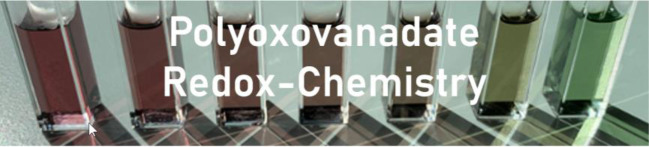

Molecular metal oxides, so‐called polyoxometalates (POM) are unique functional molecules which have attracted immense interest due to their tunable structure and reactivity.^[1]^ POMs have been used in diverse areas of science and technology, ranging from biomedicine and therapeutics[^2^, ^3^, ^4^] to molecular electronics,[^5^, ^6^] supramolecular assemblies[^7^, ^8^] and catalysis.^[9]^

One key function of POMs is their ability to reversibly store and release multiple electrons, making them ideal molecular components for energy conversion and storage technologies.[^10^, ^11^] In general, each electron added to a POM cluster shifts the reduction potential to more negative values.^[12]^ The electrochemical potential difference between two reversible one‐electron transitions in POMs is commonly in the range of ~0.4 V to 0.6 V (see SI, Section 2.11). In addition, the maximum overall number of electrons which can be stored per cluster unit is controlled by the cluster type (i.e. the number and type of metal centers present), as well as the electrochemical stabilities of the cluster, the solvent and the supporting electrolyte. For most applications in energy storage (batteries, redox‐flow batteries, solar energy storage, *etc*.), increasing the number of electrons stored is a major development driver.[^13^, ^14^]

Inspired by these challenges, ground‐breaking works have focused on how to maximize electron storage in POMs. Irle, Yoshikawa, Awaga and co‐workers showed that POM integration into lithium ion battery electrodes allows reversible storage of up to 24 electrons on the Keggin polyoxotungstate [PMo_12_O_40_]^n−^ (*n*=3–27).[^17^, ^18^] Pioneering work on the solution redox chemistry of POMs by Symes, Poblet, Nyman and Cronin showed that super‐reduced Dawson polyoxotungstates [P_2_W_18_O_62_]^n−^ (*n*=6–24) can reversibly store up to 18 electrons per cluster in aqueous media in the presence of charge‐balancing Li^+^ or H^+^ counterions. The system was further developed for electrochemical on‐demand hydrogen generation.[^19^, ^20^]

Polyoxovanadates (POVs) have recently become a focal point for multi‐electron storage due to their unique structural and electronic versatility[^21^, ^22^, ^23^, ^24^, ^25^] combined with exceptional redox properties.[^11^, ^26^, ^27^] In a series of outstanding reports, Matson and co‐workers showed that derivatives of alkoxide‐functionalized Lindqvist POVs [V_6_O_7_(OR)_12_] (R=Me, Et) can undergo multiple (proton‐coupled) electron transfers, enabling energy storage in non‐aqueous POV‐based redox‐flow batteries.[^28^, ^29^, ^30^] Also, some of us recently showed how metal‐functionalization in dodecavanadate derivatives can be used to tune the number and position of redox‐transitions.[^31^, ^32^, ^33^] While these studies were primarily focused on energy technologies, it is worth noting that mixed‐valent POVs have also received widespread interest from the molecular magnetism and spintronics communities as potential components for data storage systems. When considering the use of POMs in multi‐electron storage and transport, one common theme is that these studies typically employ only one POM cluster species at a time. In contrast, the combined use of two or more species to maximize electron storage capacity has not been explored to date. This might be due to challenges related to incompatibilities between species including inter‐cluster redox‐reactions, divergent chemical stability or solubility ranges.

Here, we propose a concept to overcome these challenges by developing designer electron storage materials where two individual POV clusters are synthesized and selectively crystallized from one common reaction solution. Importantly, cluster formation and redox equilibration are achieved within the reaction solution, thereby ensuring that no chemical incompatibilities arise for the two targeted cluster species. This approach results in a 1 : 1 mixture of two POVs with the formal assembly shown in Figure 1, where the combination of individual redox‐processes at both clusters results in up to fourteen redox‐transitions in the potential range between −2.15 V to +1.35 V (vs Fc^+^/Fc). Mechanistic studies rationalize cluster formation, cluster stability and charge‐storage mechanism. These insights shed light on how physical and electronic structure interact to give rise to technologically important redox properties.

**Figure 1:**
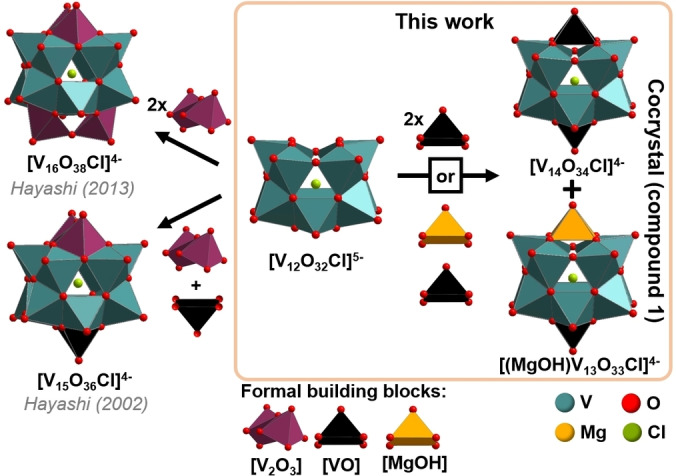
Formal assembly Scheme and structure comparison of the mixed‐cluster cocrystal **1**, compared with structurally related literature‐known single‐cluster species (*n*Bu_4_)_4_[V_16_O_38_Cl] and (*n*Bu_4_)_4_[V_15_O_36_Cl].[^15^, ^16^] Note that synthetically, **1** is obtained using [V_10_O_26_]^4−^ as vanadate precursor.

To develop the mixed‐cluster system, we built on previous studies by some of us, where we explored the mono‐ or di‐metal‐functionalization of the dodecavanadate cluster (NMe_2_H_2_)_2_[V_12_O_32_Cl]^3−^ (=**{V_12_}**).[^33^, ^34^, ^35^, ^36^, ^37^] Here, we demonstrate how under non‐aqueous synthesis conditions, simultaneous formation and redox equilibration of two closely related **{V_12_}** derivatives was achieved, This gave access to mixed co‐crystals showing reversible multi‐electron uptake and release.

The title compound **1** was synthesized by reaction of the mixed‐valent precursor (*n*Bu_4_N)_4_[V^IV^_2_V^V^_8_O_26_] (=(*n*Bu_4_N)_4_ **{V_10_}**)[^38^, ^39^] with the magnesium and chloride source MgCl_2_ (anhydrous) in acetonitrile at 75 °C. Diffusion of diethyl ether into the green reaction solution gave deep green crystals of **1** suitable for single‐crystal X‐ray diffraction (yield: 70 % based on V). The composition and purity of the compound were confirmed by ATR‐IR‐ UV/Vis/NIR and EPR spectroscopies, high‐resolution electrospray ionization mass spectrometry (ESI MS), thermogravimetric analysis (TGA) and single‐crystal X‐ray diffraction, for details see Supporting Information.

Single‐crystal X‐ray diffraction analysis showed that **1** crystallizes in the monoclinic space group *C*_2_ with cell axes *a*=23.189(4) Å, *b*=23.197(4) Å, *c*=17.660(3) Å and angles α=γ=90°, *β*=90.089(6), crystallographic details see SI, Section 3.^[40]^

Structural analysis of the crystallographic data reveal that **1** is composed of a 1 : 1 mixture of two **{V_12_}** derivatives, that is the mixed magnesium and vanadium/functionalized species [(MgOH)V_13_O_33_Cl]^4−^ (=**{MgV_13_}**) and the di‐vanadium‐functionalized species [V_14_O_34_Cl]^4−^ (=**{V_14_}**). Note that to the best of our knowledge, both clusters, **{MgV_13_}** and **{V_14_}** have not been reported previously. For charge‐balance, each unit cell contains two cluster units and eight *n*Bu_4_N^+^ cations. The amount of *n*Bu_4_N^+^ cations in the bulk was verified by CHN elemental analysis and TGA (see SI, Section 2.3). The **{V_14_}** : **{MgV_13_}** molar ratio in the bulk of **1** was analyzed by inductively coupled plasma optical emission spectroscopy (ICP‐OES), which gave a Mg : V atomic ratio of 1.17: 27 (calculated: 1.0 : 27.0 for a 1 : 1 molar ratio of **{MgV_13_}** and **{V_14_}**). The presence of the Mg−OH group in **1** was substantiated by ATR‐IR spectroscopy, where a weak, characteristic signal was detected at 3460 cm^−1^ (Figure 2a).^[41]^ The presence of both cluster species, **{MgV_13_}** and **{V_14_}**, was also observed by negative‐ion mode high‐resolution electrospray ionization mass spectrometry (ESI MS), see Figure 2c,d. For example, the **{MgV_13_}**‐related species (*n*Bu_4_N)[HMgV_12_O_32_Cl]^−^ was observed at 1667.69 m/z (calcd: 1667.73 m/z), while the **{V_14_}** species (*n*Bu_4_N)[V_14_O_34_Cl]^2−^ was observed at 767.15 m/z (calcd: 767.17 m/z)

**Figure 2:**
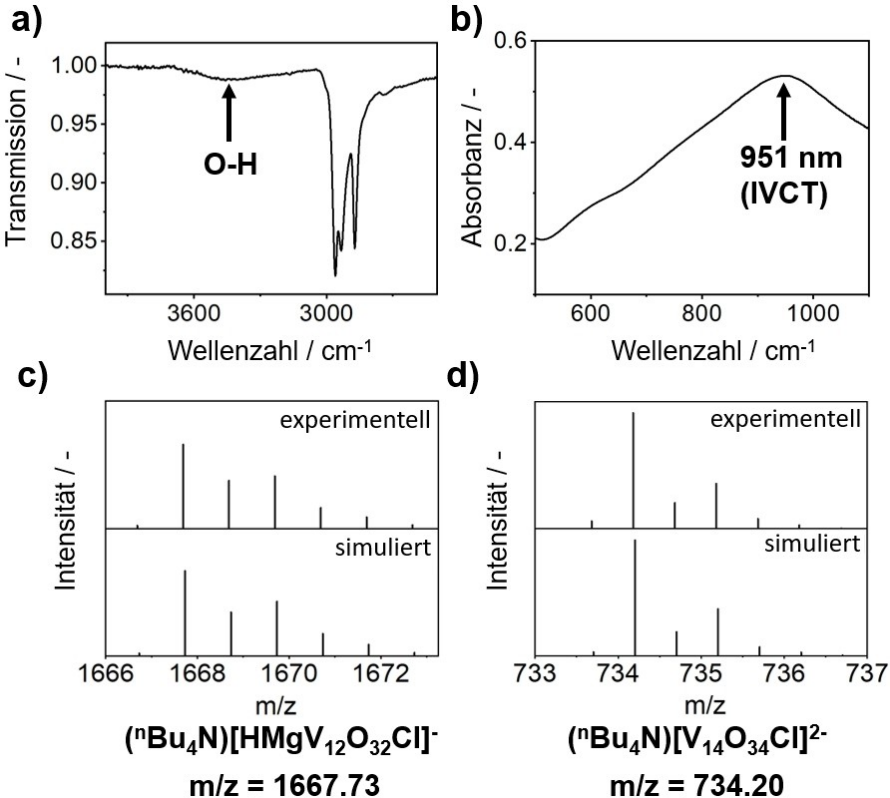
Characterization of **1**. a) ATR‐IR spectroscopy showing the presence of O−H vibrations; b) UV/Vis/NIR spectroscopy showing characteristic V(IV/V) intervalence charge‐transfer (IVCT) transitions; c), d) simulated and observed isotopic patterns from high resolution ESI MS analyses, indicating the presence of **{MgV_13_}** and **{V_14_}**.

In sum, these analyses suggest that **1** can be given as (*n*Bu_4_N)_8_[(MgOH)V_13_O_33_Cl][V_14_O_34_Cl]=(*n*Bu_4_N)_8_ **{MgV_13_}{V_14_}**.

Based on this initial analysis it can be proposed that each cluster features a fourfold negative charge. This would result in three V^IV^ centers for **{MgV_13_}**, and five V^IV^ centers for **{V_14_}**, for details on the charge calculations see SI, Section 2.4. Since most mixed‐valent POMs feature antiferromagnetic coupling,[^5^, ^23^, ^42^, ^43^, ^44^] it can be put forward that both, **{MgV_13_}** and **{V_14_}** feature one unpaired electron. Thus, the presence of unpaired electrons in **1** was probed by EPR spectroscopy (Figure 3). As a reference compound for spin counting, we utilized the mixed‐valent precursor (*n*Bu_4_N)_4_ **{V_10_}**[^38^, ^39^] which features two isolated V^IV^ centers. Note that only the EPR spectrum of the reference compound shows hyperfine coupling, as electron delocalization in **1** results in a non‐observable hyperfine coupling.^[45]^ A comparison of the spin count of both compounds at the same concentration and same sample volume, gave 1.87 unpaired electrons per formula unit of **1**. This is in line with our previous oxidation state and charge assignments which suggested that both **{MgV_13_}** and **{V_14_}** feature one unpaired electron each. In addition, UV/Vis/NIR spectroscopy of **1** in MeCN also supported the presence of reduced V^IV^ centers as indicated by characteristic intervalence charge transfer (IVCT) transitions in the ~500 nm – 1400 nm spectral region (Figure 2b).

**Figure 3:**
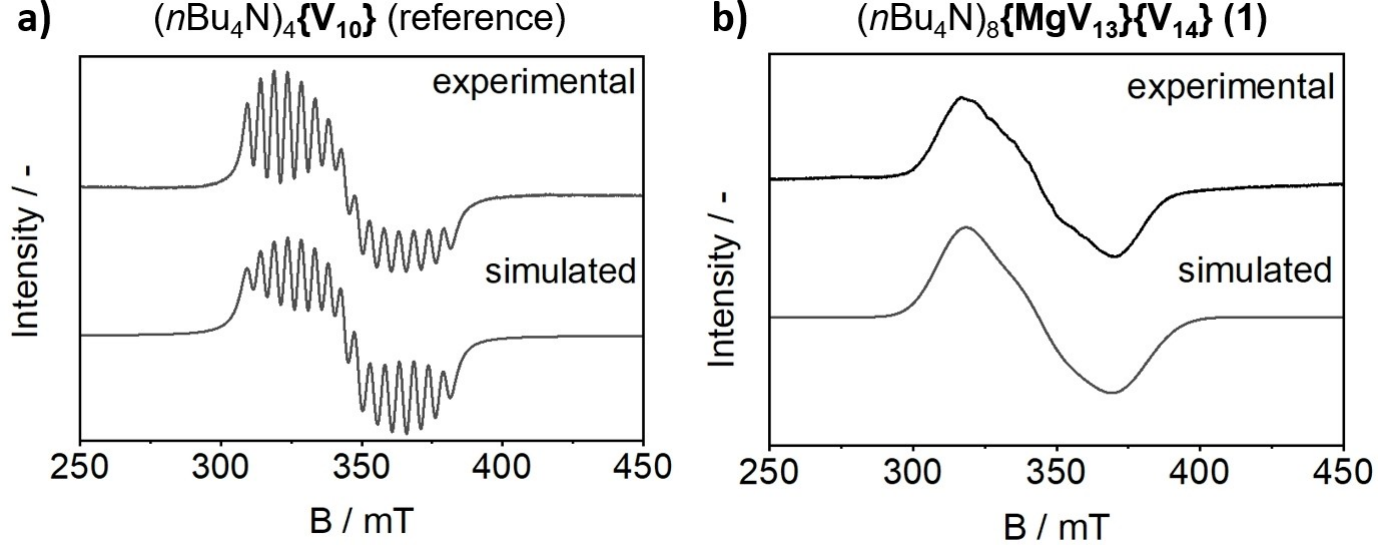
Simulated and experimental EPR spectra (*T*=40 °C) of: a) the reference compound (*n*Bu_4_N)_4_[V^IV^_2_V^V^_8_O_24_]^[38]^ (3 mM in MeCN); b) compound **1** (3 mM in MeCN). Comparison of the spin counts gave a value of 1.87 unpaired electrons for **1**.

We were interested in the Mg^2+^‐induced conversion of the mixed‐valent **{V_10_}** precursor into compound **1**. Note that in the absence of MgCl_2_, no conversion of **{V_10_}** into **1** occurs. We used UV/Vis/NIR spectroscopy to follow the changes of the characteristic signals of **{V_10_}** (at λ=500 nm) and **1** (at λ=1000 nm), see Figure 4a. We hypothesized that the reaction might be initiated by reaction of Mg^2+^ with **{V_10_}**, possibly by a Lewis‐acid mechanism. Thus, we investigated the impact of **{V_10_}**/Mg^2+^ molar ratios on the conversion rate by a series of [Mg^2+^]‐dependent analyses. As shown in Figure 4, between 0–1 molar equivalents of Mg^2+^ (relative to **{V_10_}**), increasing Mg^2+^ concentrations result in equilibria with decreasing **{V_10_}** concentration (Figure 4b) and increasing concentrations of **1** (Figure 4c). When more than ~1 equivalent of Mg^2+^ is added to the reaction solution, the characteristic UV/Vis/NIR spectral signatures of **{V_10_}** are lost, and no further changes of the characteristic signatures of **1** are observed. These data suggest that the **{V_10_}** conversion to **1** is driven by the presence of Mg^2+^. Previous studies have reported similar observations, where Lewis‐acidic metals, such as Mg^2+^,^[36]^ Ca^2+^,[^32^, ^33^] Co^2+^, Ti^4+^ or Cu^2+[46]^ have triggered conversion processes in polyoxo‐vanadates, particularly in organic solvents.

**Figure 4:**
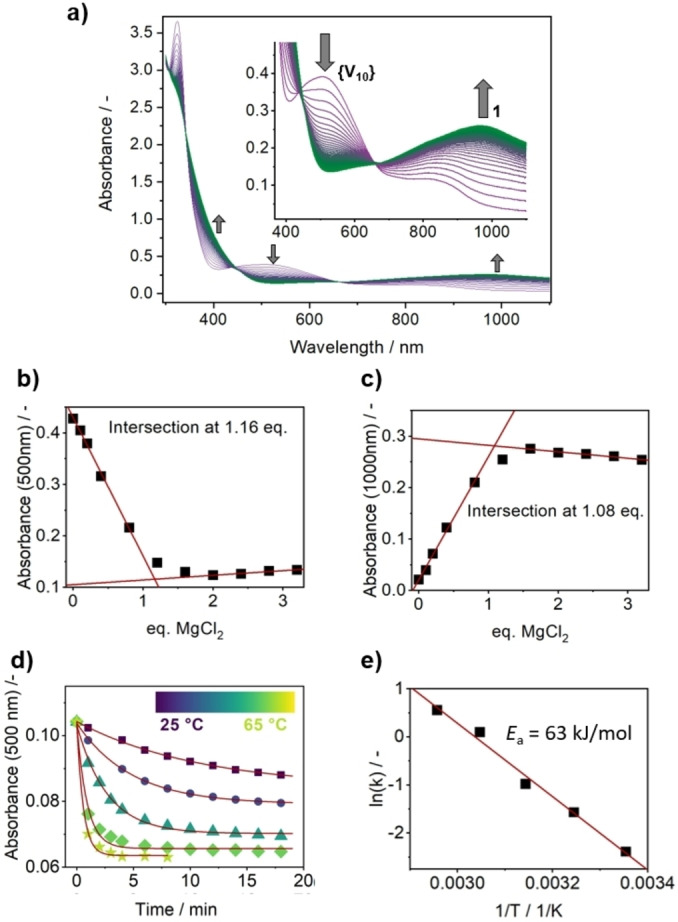
a) Time‐lapse UV/Vis/NIR spectroscopy of the conversion of **{V_10_}** to **1** at time intervals of 1 min. Three isosbestic points at λ_1_=665 nm, λ_2_=446 nm and λ_3_=340 nm indicate a direct conversion process. Conditions: *T*=35 °C, cuvette path length 10.0 mm, [**{V_10_}**] : [MgCl_2_ (anhydrous)]=1 : 1.2, [**{V_10_}**]=230 μM). b) Decrease of **{V_10_}** concentration as a function of [Mg^2+^] concentration, monitored by following the characteristic **{V_10_}** absorbance at λ=500 nm; samples were equilibrated for 1 h before measurement; c) Increase of the concentration of **1** as a function of Mg^2+^ concentration, monitored by following the characteristic absorbance of **1** at λ=1000 nm; samples were equilibrated for 1 h before measurement. d) Kinetic traces and mono‐exponential fits of the **{V_10_}**‐to‐**1** conversion (monitored by following the decrease of **{V_10_}** concentration at λ=500 nm) at temperatures between 25 °C to 65 °C. Conditions: Δ*T*=10 °C, cuvette path length 2.0 mm, [**{V_10_}**] : [MgCl_2_ (anhydrous)]=1 : 2.2, [**{V_10_}**]=500 μM. d) Arrhenius plot based on the kinetic traces shown in e).

Thus, we determined the apparent conversion rate constant *k* at temperatures between 25 °C – 65 °C to provide initial thermochemical data for the **{V_10_}**‐to‐**1** conversion. As illustrated in Figure 4d, this allowed an Arrhenius‐type plot showing linear behaviour, which gave an activation energy for the conversion of 63 kJ/mol.

Next, we explored the electrochemistry of **1**, as we hypothesized that the presence of both, **{MgV_13_}** and **{V_14_}** clusters in **1** could result in enhanced redox‐reactivity and electron storage capacity. To prevent any effects of surface oxidation of the crystalline sample (which was suggested by microscopic analysis of the native crystals after storage in air), we purified **1** by re‐crystallization from acetonitrile under inert atmosphere and water‐free conditions in an Ar‐filled glovebox (see SI, Section 2.1). These samples were used for all electrochemical and EPR studies. Electrochemistry was performed in inert Ar atmosphere in a glovebox (for details see SI, Sections 2.11 and 2.12).

Initial electrochemical analyses of **1** (0.5 mM) were performed by cyclic voltammetry (CV) and square wave voltammetry (SWV) in anhydrous, degassed acetonitrile solution containing *n*Bu_4_NPF_6_ (0.1 M) as supporting electrolyte (Figure 5). All electrochemical data were recorded using a silver/silver nitrate reference electrode, and the potentials were then referenced against an internal ferrocene/ferrocenium (Fc^+^/Fc) redox couple. In the potential range between −2.15 V and +1.35 V, **1** showed up fourteen redox transitions (see green labels in Figure 5). Integration of the complex SWV data results in the tentative assignment that twelve processes (processes 1, 2, 5–14) are 1‐electron transfers, while two processes (processes 3, 4) are 2‐electron transfers (see SI, Section 2.12). In sum, this suggests uptake/release of 16 electrons by **1** in the given potential range. Note that initial density functional theory (DFT) computations for the first reduction of **{V_14_}** and **{MgV_13_}** gave theoretical redox potentials which are in close agreement (Δ*E*<5 mV) with the experimentally observed data (i.e., redox‐transitions 8 and 9). For details see SI, Section 2.15.

**Figure 5:**
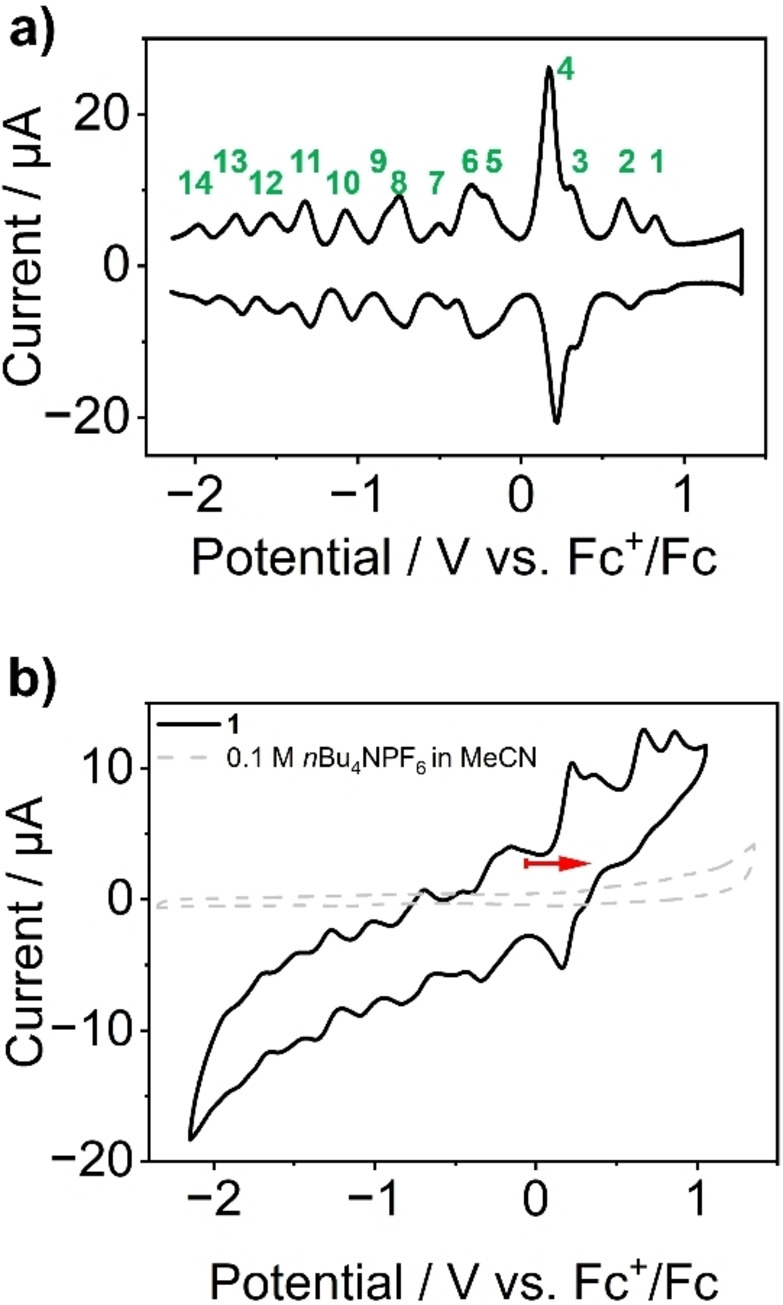
a) Square wave voltammogram and b) cyclovoltammogram of **1**, showing 14 pseudo‐reversible redox transitions in the scan range between −2.15 V to+1.35 V vs Fc+/Fc, open circuit potential and scan direction are indicated by the red arrow. Conditions: anhydrous, deoxygenated acetonitrile containing *n*Bu_4_NPF_6_ (0.1 M) as supporting electrolyte. Scan rate 50 mV s^−1^), [**1**]: 0.5 mM, Ar atmosphere.

To verify these findings and assess the bulk redox activity of **1**, we performed bulk electrolysis (BE) under the conditions used for CV and SWV. Under reductive BE conditions (*E*_red_=‐ 1.45 V), the data show that 7.86±0.82 electrons can be taken up per formula unit of **1** (i.e., per one **{MgV_13_}** and one **{V_14_}** cluster) when starting from the native compound. Note that no significant changes of the SWV data are observed upon bulk reduction, indicating that the structural integrity of the clusters are retained (SI, Section 2.12). Also, an increase of the characteristic IVCT transitions in the VIS‐NIR range are in line with the increasing number of V^IV^ centers. Under oxidative conditions (*E*_ox_=+1.22 V), BE resulted in the release of 8.66±0.86 electrons per formula unit of **1**. This is in line with a four‐electron oxidation of the **{MgV_13_}** and **{V_14_}** species, respectively, resulting in the complete removal of all V^IV^ centers. This is supported by UV/Vis‐NIR spectroscopy of the oxidized sample of **1**, where a complete loss of the IVCT transitions in the VIS‐NIR range (characteristic of delocalized V^IV^ centers) was observed (SI, Section 2.12, Figure S14). Note that for the fully oxidized sample, the SWV signatures became somewhat less well defined compared to the native sample. However, upon re‐reduction of the sample at the open‐circuit potential OCP (*E*_OCP_=‐0.06 V vs. Fc^+^/Fc), the original SWV signals of **1** were recovered (SI, Section 2.12, Figure S15), suggesting that the structural integrity in this sample is retained or regenerated upon re‐reduction. In sum BE analyses indicate that reversible uptake and release of up to 16 electrons per formula unit of **1** is possible in the potential range between ‐ 1.08 V to 1.07 V vs. Fc/Fc^+^ (for details see SI, Section 2.12). This is in line with the data observed from integration of the SWV data, where 16 electron transfers were estimated (Figure 5 and SI, Section 2.11). Note that the physical mixing of two chemically or structurally related polyoxovanadates leads to complex mixtures where redox reactions between the clusters can occur, which can lead to (partial) degradation or cluster rearrangements. Thus, redox equilibration during synthesis is an effective path to avoid this detrimental reaction (see SI, Figure S17).

In sum, we have shown that redox‐equilibration of structurally related but chemically different polyoxovanadate clusters is a new and viable synthetic route to access electroactive compounds with remarkable reversible redox‐reactivity. Specifically, self‐assembly and co‐crystallization of two vanadium oxide clusters, **{MgV_13_}** and **{V_14_}** results in an unusual system where the redox‐events of the two species facilitate closely aligned redox‐transitions, so that up to 16 electrons can be stored in a potential range of ~3.5 V. In future, this new materials design approach will be used to develop high‐performance electrolytes for non‐aqueous redox‐flow batteries.

## Supporting Information

The authors have cited additional references within the Supporting Information.[^47^, ^48^, ^49^, ^50^, ^51^, ^52^, ^53^, ^54^, ^55^, ^56^, ^57^, ^58^, ^59^, ^60^, ^61^, ^62^, ^63^, ^64^, ^65^]

## Conflict of Interests

The authors declare no conflict of interest.

## Supporting information

As a service to our authors and readers, this journal provides supporting information supplied by the authors. Such materials are peer reviewed and may be re‐organized for online delivery, but are not copy‐edited or typeset. Technical support issues arising from supporting information (other than missing files) should be addressed to the authors.

Supporting Information

Supporting Information

## Data Availability

The data that support the findings of this study are openly available in Zenodo.org at 10.5281/zenodo.13002159, reference number 13002159.

## References

[1] L. Cronin, A. Müller, (guest eds.), special POM themed issue, *Chem. Soc. Rev* **2012**, *41*, 7325–7648.

[2] L. S. Van Rompuy , T. N. Parac-Vogt , Curr. Opin. Biotechnol. 2019, 58, 92–99. 30529815 10.1016/j.copbio.2018.11.013

[3] F. de Azambuja , J. Moons , T. N. Parac-Vogt , Acc. Chem. Res. 2021, 54, 1673–1684. 33600141 10.1021/acs.accounts.0c00666

[4] A. Bijelic , M. Aureliano , A. Rompel , Angew. Chem. Int. Ed. 2019, 58, 2980–2999. 10.1002/anie.201803868 PMC6391951 29893459

[5] M. Moors , J. Warneke , X. Lopez , C. de Graaf , B. Abel , K. Y. Monakhov , Acc. Chem. Res. 2021, 54, 3377–3389. 34427081 10.1021/acs.accounts.1c00311

[6] E. Coronado , Nat. Rev. Mater. 2019, 5, 87–104.

[7] M. Stuckart , K. Y. Monakhov , Chem. Sci. 2019, 10, 4364–4376. 31057763 10.1039/c9sc00979e PMC6482875

[8] A. Misra , K. Kozma , C. Streb , M. Nyman , Angew. Chem. Int. Ed. 2020, 59, 596–612. 10.1002/anie.201905600 PMC6972580 31260159

[9] S. Wang , G. Yang , Chem. Rev. 2015, 115, 4893–4962. 25965251 10.1021/cr500390v

[10] A. Sartorel , M. Bonchio , S. Campagna , F. Scandola , Chem. Soc. Rev. 2013, 42, 2262–2280. 23011384 10.1039/c2cs35287g

[11] M. Anjass , G. A. Lowe , C. Streb , Angew. Chem. Int. Ed. 2021, 60, 7522–7532. 10.1002/anie.202010577 PMC8048609 32881270

[12] T. Ueda , ChemElectroChem 2018, 5, 823–838.

[13] D. G. Nocera , J. Am. Chem. Soc. 2022, 144, 1069–1081. 35023740 10.1021/jacs.1c10444

[14] Z. Li , Y. C. Lu , Adv. Mater. 2020, 32, 2002132.

[15] Y. Hayashi , N. Miyakoshi , T. Shinguchi , A. Uehara , Chem. Lett. 2001, 30, 170–171.

[16] N. Kato , Y. Hayashi , Dalton Trans. 2013, 42, 11804–11811. 23857565 10.1039/c3dt50521a

[17] Y. Nishimoto , D. Yokogawa , H. Yoshikawa , K. Awaga , S. Irle , J. Am. Chem. Soc. 2014, 136, 9042–9052. 24885348 10.1021/ja5032369

[18] H. Wang , S. Hamanaka , Y. Nishimoto , S. Irle , T. Yokoyama , H. Yoshikawa , K. Awaga , J. Am. Chem. Soc. 2012, 134, 4918–4924. 22352694 10.1021/ja2117206

[19] J. J. Chen , M. D. Symes , L. Cronin , Nat. Chem. 2018, 10, 1042–1047. 30104721 10.1038/s41557-018-0109-5

[20] J.-J. Chen , L. Vilà-Nadal , A. Solé-Daura , G. Chisholm , T. Minato , C. Busche , T. Zhao , B. Kandasamy , A. Y. Ganin , R. M. Smith , I. Colliard , J. J. Carbó , J. M. Poblet , M. Nyman , L. Cronin , J. Am. Chem. Soc. 2022, 144, 8951–8960. 35536652 10.1021/jacs.1c10584 PMC9171825

[21] C. Streb, “Structure and Bonding in Molecular Vanadium Oxides: From Templates via Host–Guest Chemistry to Applications.” in: Polyoxometalate-Based Assemblies and Functional Materials. Structure and Bonding, Vol 176 (guest ed: Y.-F. Song). Springer, Cham, **2017**, pp. 31–47.

[22] K. Y. Monakhov, M. Moors, P. Kögerler, “Perspectives for Polyoxometalates in Single-Molecule Electronics and Spintronics“, in *Advances in Inorganic Chemistry*, Vol. 69 (Eds.: R. van Eldik, L. Cronin), Academic Press, Amsterdam, **2017**, pp. 251–286.

[23] K. Yu Monakhov , W. Bensch , P. Kögerler , Chem. Soc. Rev. 2015, 44, 8443–8483. 26344788 10.1039/c5cs00531k

[24] D. Gatteschi , L. Pardi , A. L. Barra , A. Müller , Molec. Engin. 1993, 3, 157–169.

[25] W. G. Klemperer , T. A. Marquart , O. M. Yaghi , Angew. Chem. Int. Ed. Engl. 1992, 31, 49–51.

[26] S. Chakraborty , B. E. Petel , E. Schreiber , E. M. Matson , Nanoscale Adv. 2021, 3, 1293–1318. 36132875 10.1039/d0na00877j PMC9419539

[27] K. R. Proe , E. Schreiber , E. M. Matson , Acc. Chem. Res. 2023, 2021, 12. 10.1021/acs.accounts.3c00166 PMC10286311 37279252

[28] L. E. VanGelder , T. R. Cook , E. M. Matson , Comm. Inorg. Chem. 2019, 39, 51–89.

[29] L. E. VanGelder , A. M. Kosswattaarachchi , P. L. Forrestel , T. R. Cook , E. M. Matson , Chem. Sci. 2018, 9, 1692–1699. 29675217 10.1039/c7sc05295b PMC5890794

[30] L. E. VanGelder , E. M. Matson , J. Mater. Chem. A 2018, 6, 13874–13882.

[31] M. H. Anjass , K. Kastner , F. Nägele , M. Ringenberg , J. F. Boas , J. Zhang , A. M. Bond , T. Jacob , C. Streb , Angew. Chem. Int. Ed. 2017, 56, 14749–14752. 10.1002/anie.201706828 28906058

[32] S. Greiner , B. Schwarz , C. Streb , M. Anjass , Chem. Eur. J. 2021, 27, 13435–13441. 34288174 10.1002/chem.202102352 PMC8519020

[33] S. Greiner , B. Schwarz , M. Ringenberg , M. Dürr , I. Ivanovic-Burmazovic , M. Fichtner , M. Anjass , C. Streb , Chem. Sci. 2020, 11, 4450–4455. 34122902 10.1039/d0sc01401j PMC8159454

[34] K. Kastner , J. T. Margraf , T. Clark , C. Streb , Chem. Eur. J. 2014, 20, 12269–12273. 25082170 10.1002/chem.201403592

[35] K. Kastner , J. Forster , H. Ida , G. N. Newton , H. Oshio , C. Streb , Chem. Eur. J. 2015, 21, 7686–7689. 25850969 10.1002/chem.201501049

[36] S. Repp , M. Remmers , A. Stefanie , J. Rein , D. Sorsche , D. Gao , M. Anjass , M. Mondeshki , L. M. Carrella , E. Rentschler , C. Streb , Nat. Commun. 2023, 14, 5563. 37689696 10.1038/s41467-023-41257-y PMC10492840

[37] N. Arya , T. Philipp , S. Greiner , M. Steiner , C. Kranz , M. Anjass , Angew. Chem. Int. Ed. 2023, 62, e202306170. 10.1002/anie.202306170 37218398

[38] A. Bino , S. Cohen , C. Heitner-Wirguin , Inorg. Chem. 1982, 21, 429–431.

[39] K. Okaya , T. Kobayashi , Y. Koyama , Y. Hayashi , K. Isobe , Eur. J. Inorg. Chem. 2009, 2009, 5156–5163.

[40] Deposition Number 2369209 (for **1**)) contains the supplementary crystallographic data for this paper. These data are provided free of charge by the joint Cambridge Crystallographic Data Centre and Fachinformationszentrum Karlsruhe Access Structures service.

[41] A. Kondo , R. Kurosawa , J. Ryu , M. Matsuoka , M. Takeuchi , J. Phys. Chem. C 2021, 125, 10937–10947.

[42] P. Kögerler , B. Tsukerblat , A. Müller , Dalton Trans. 2010, 39, 21–36. 10.1039/b910716a 20023927

[43] A. Müller , P. Kögerler , A. W. M. Dress , Coord. Chem. Rev. 2001, 222, 193–218.

[44] J. Wang , C. Näther , P. Kögerler , W. Bensch , Inorg. Chim. Acta 2010, 363, 4399–4404.

[45] O. Linnenberg , P. Kozlowski , C. Besson , J. Van Leusen , U. Englert , K. Y. Monakhov , Cryst. Growth Des. 2017, 17, 2342–2350.

[46] T. Kobayashi , S. Kuwajima , T. Kurata , Y. Hayashi , Inorg. Chim. Acta 2014, 420, 69–74.

[47] Y. Cao , J.-J. J. Chen , M. A. Barteau , J. Energy Chem. 2020, 50, 115–124.

[48] L. E. VanGelder , A. M. Kosswattaarachchi , P. L. Forrestel , T. R. Cook , E. M. Matson , Chem. Sci. 2018, 9, 1692–1699. 29675217 10.1039/c7sc05295b PMC5890794

[49] N. Fay , A. M. Bond , C. Baffert , J. F. Boas , J. R. Pilbrow , D.-L. Long , L. Cronin , Inorg. Chem. 2007, 46, 3502–3510. 17391025 10.1021/ic062067e

[50] S. Greiner , B. Schwarz , M. Ringenberg , M. Dürr , I. Ivanovic-Burmazovic , M. Fichtner , M. Anjass , C. Streb , Chem. Sci. 2020, 11, 4450–4455. 34122902 10.1039/d0sc01401j PMC8159454

[51] O. Linnenberg , M. Moors , A. Solé-Daura , X. López , C. Bäumer , E. Kentzinger , W. Pyckhout-Hintzen , K. Yu Monakhov , J. Phys. Chem. C 2017, 121, 10419–10429.

[52] G. M. Sheldrick , Acta Crystallogr. Sect. A 2008, 64, 112–122. 18156677 10.1107/S0108767307043930

[53] O. V. Dolomanov , L. J. Bourhis , R. J. Gildea , J. A. K. Howard , H. Puschmann , J. Appl. Crystallogr. 2009, 42, 339–341. 10.1107/S0021889811041161 PMC3236671 22199401

[54] L. J. Bourhis , O. V. Dolomanov , R. J. Gildea , J. A. K. Howard , H. Puschmann , Acta Crystallogr. Sect. A 2015, 71, 59–75. 10.1107/S2053273314022207 PMC4283469 25537389

[55] R. H. Blessing , Acta Crystallogr. Sect. A 1995, 51, 33–38. 7702794 10.1107/s0108767394005726

[56] T. Ueda , K. Kodani , H. Ota , M. Shiro , S.-X. Guo , J. F. Boas , A. M. Bond , Inorg. Chem. 2017, 56, 3990–4001. 28290689 10.1021/acs.inorgchem.6b03046

[57] R. L. Belford , N. D. Chasteen , H. So , R. E. Tapscott , J. Am. Chem. Soc. 1969, 91, 4675–4680.

[58] Q. Chen , D. P. Goshorn , C. P. Scholes , X. L. Tan , J. Zubieta , J. Am. Chem. Soc. 1992, 114, 4667–4681.

[59] S. Stoll , A. Schweiger , J. Magnet. Res. 2006, 178, 42–55. 10.1016/j.jmr.2005.08.013 16188474

[60] K. J. Laidler , J. Chem. Educ. 1984, 61, 494.

[61] V. W. Day , W. G. Klemperer , O. M. Yaghi , J. Am. Chem. Soc. 1989, 111, 5959–5961.

[62] V. W. Day , W. G. Klemperer , D. J. Maltbie , J. Am. Chem. Soc. 1987, 109, 2991–3002.

[63] A. V. Marenich , C. J. Cramer , D. G. Truhlar , J. Phys. Chem. B 2009, 113, 6378–6396. 19366259 10.1021/jp810292n

[64] F. Weigend , R. Ahlrichs , Phys. Chem. Chem. Phys. 2005, 7, 3297. 16240044 10.1039/b508541a

[65] Gaussian 16, Revision C.01, M. J. Frisch, G. W. Trucks, H. B. Schlegel, G. E. Scuseria, M. A. Robb, J. R. Cheeseman, G. Scalmani, V. Barone, G. A. Petersson, H. Nakatsuji, X. Li, M. Caricato, A. V. Marenich, J. Bloino, B. G. Janesko, R. Gomperts, B. Mennucci, H. P. Hratchian, J. V. Ortiz, A. F. Izmaylov, J. L. Sonnenberg, D. Williams-Young, F. Ding, F. Lipparini, F. Egidi, J. Goings, B. Peng, A. Petrone, T. Henderson, D. Ranasinghe, V. G. Zakrzewski, J. Gao, N. Rega, G. Zheng, W. Liang, M. Hada, M. Ehara, K. Toyota, R. Fukuda, J. Hasegawa, M. Ishida, T. Nakajima, Y. Honda, O. Kitao, H. Nakai, T. Vreven, K. Throssell, J. A. Montgomery, Jr., J. E. Peralta, F. Ogliaro, M. J. Bearpark, J. J. Heyd, E. N. Brothers, K. N. Kudin, V. N. Staroverov, T. A. Keith, R. Kobayashi, J. Normand, K. Raghavachari, A. P. Rendell, J. C. Burant, S. S. Iyengar, J. Tomasi, M. Cossi, J. M. Millam, M. Klene, C. Adamo, R. Cammi, J. W. Ochterski, R. L. Martin, K. Morokuma, O. Farkas, J. B. Foresman, and D. J. Fox, Gaussian, Inc., Wallingford CT, 2016.

