# Supplementary material for: Redox Self‐Equilibration in Molecular Vanadium Oxide Mixtures Enables Multi‐Electron Storage
Source: Angew Chem Int Ed Engl. 2024 Nov 16;64(2):e202418864. doi: 10.1002/anie.202418864 (PMC11720375; doi:10.1002/anie.202418864)
Supplement: Supplementary file 2 — Supporting Information [file ANIE-64-e202418864-s001.pdf]

Angewandte Chemie
Eine Zeitschrift der Gesellschaft Deutscher Chemiker

Supporting Information

Redox Self-Equilibration in Molecular Vanadium Oxide Mixtures Enables Multi-Electron Storage

*M. Remmers, B. Mashtakov, S. Repp, A. S. J. Rein, K. Wang, M. Anjass, Z. Chen, L. M. Carrella, E. Rentschler, C. Streb**

Supporting Information:

1. Instrumentation: ... 2
2. Experimental data ... 3
   2.1. Synthesis of (nBu$_4$N)$_8$[V$_{14}$O$_{34}$Cl][(MgOH)V$_{13}$O$_{33}$Cl] (Compound 1) ... 3
   2.1. ^{1}H-NMR Spectroscopy ... 4
   2.2. EPR Spectroscopy ... 4
   2.3. Thermogravimetric Analysis ... 5
   2.4. Cluster Charge Calculations ... 6
   2.5. ESI Mass Spectrometry ... 7
   2.6. UV/Vis/NIR Spectroscopy ... 8
   2.7. Temperature dependent UV/Vis/NIR Spectroscopy ... 9
   2.8. Determination of the Molar Activation Energy ... 10
   2.9. Mg^{2+} Concentration-dependent UV/Vis/NIR ... 11
   2.10. ATR-FT-IR spectroscopy ... 12
   2.11. Electrochemistry ... 13
   2.12. Bulk Electrolysis ... 14
   2.13. Crystallographic Information ... 18
   2.14. Electrochemical reference studies ... 19
   2.15. Computational Section ... 19
3. References ... 20

1. Instrumentation:

Single-crystal X-ray diffraction (sc-XRD) was measured on a Bruker APEX-II CCD Single-crystal X-ray diffractometer equipped with a graphite monochromator using Mo Kα radiation (wavelength λ(Mo Kα) = 0.71073 Å)

Thermogravimetric analysis: (TGA) was performed on METTLER TOLEDO TGA 2 STARe system under air flow with a flow rate of 60 mL min^{-1}. A polycrystalline aluminium oxide crucible (PCA) was used. Samples were analyzed in a temperature range of 303.15−1173.15 K. A heating rate of 10 K min^{-1} was applied.

Attenuated total reflectance-Fourier-transformed infrared spectroscopy (ATR-FT-IR) were performed using a Bruker Alpha II equipped with an ATR Platinum Diamond unit. The data were recorded with 24 scans at a resolution of 4 cm^{-1}. All spectra were background-corrected within the Bruker OPUS 8.1 program suite.

^{1}H nuclear magnetic resonance (^{1}H-NMR) spectroscopy was recorded on a Bruker AVANCE Neo 400 MHz spectrometer at ambient temperature. ^{1}H NMR spectra were measured at 400 MHz. Chemical shifts value (δ) for are given in part per million (ppm) using residual solvent protons (δH = 1.94 ppm for CD_3CN).

Electron paramagnetic resonance (EPR) spectroscopy was performed on a X-band Bruker Magnettech ESR5000 spectrometer at 40 °C temperature on 5 mM acetonitrile solution of **1**. The sample was filled in a glass EPR-tube (diameter 3 mm), the measurement range was 250 to 450 mT with a modulation of 1mT, a microwave power of 100 mW and a sweep time of 120s. The SpinCount software option was used to calculate the spin quantity in the sample.

Electrochemical investigations
All experiments were carried out inside an argon-filled glovebox (MBraun LABmaster130/M200B eco) in water-free, degassed acetonitrile at room temperature, using 0.1 M $n$$Bu_4NPF_6$ as supporting electrolyte.

a) Voltammetry

Cyclic voltammetry and square wave voltammetry were performed on a CH Instruments 760E potentiostat in three-electrode configuration: a glassy carbon with d = 3.0 mm was used as working electrode, a silver wire in acetonitrile containing 0.1 M $n$$Bu_4NPF_6$ and 10 mM $AgNO_3$ was used as reference electrode and a platinum wire was used as counter electrode. All electrodes were cleaned with acetone and acetonitrile each time before use and the working electrode was additionally polished with 0.05 µm Al_2O_3 before each measurement. The recorded potentials were then referenced against the internal standard ferrocene/ferrocenium Fc^+/Fc, unless stated otherwise.

b) Bulk Electrolysis

Chronoamperometry was performed on a CH Instruments 760E potentiostat in three-electrode configuration: platinum wires were used as working and counter electrode, a silver wire in acetonitrile containing 0.1 M $n$$Bu_4NPF_6$ and 10 mM $AgNO_3$ was used as a reference electrode. All electrodes were cleaned with acetone and acetonitrile before each experiment. During the experiment the working electrode was kept at the defined potential while the solution was stirred vigorously. The electrolysis was stopped once the current dropped below 0.5 % of the initial current.

UV/Vis/NIR spectroscopy was performed on a Cary 3500 UV/Vis/NIR spectrophotometer equipped with a Xenon flash lamp (250 Hz). Measurements were performed in quartz glass cuvettes (d = 10.0 mm).

High resolution electrospray ionization mass spectrometry (ESI MS) was carried out on an Agilent 6545 QTOF-HRAM-MS system in negative ion mode at a drying gas temperature of T = 180 °C.

CHN Elemental Analysis was carried out by the central analytical service of the Department of Chemistry at Johannes Gutenberg University Mainz using an Elementar Vario EL Cube.

Inductively coupled plasma optical emission spectroscopy (ICP-OES) was performed on Agilent 5800 VDV ICP-OES (optical emission spectrometer) with the automatic sampler SPS 4. The bulk samples were diluted with 5% aqueous nitric acid.

Chemicals: All chemical reagents were obtained commercially and used as received unless stated otherwise. (nBu$_4$N)$_4$[$V^{IV}_2V^{V}_8O_{24}$] ((nBu$_4$N)$_4$**{V$_{10}$}**) was prepared according to the literature.[1]

2. Experimental data

2.1. Synthesis of (nBu$_4$N)$_8$[$V_{14}O_{34}Cl$][(MgOH)$V_{13}O_{33}Cl$] (Compound 1)

1.003 g (nBu$_4$N)$_4$**{V$_{10}$}** (0.473 mmol, 1 eq.) and 0.101 g $MgCl_2$ (1.061 mmol, 2.2 eq.) was dissolved in 25 mL acetonitrile. The solution was stirred for 4 h at 75 °C. During this period, the mixture turned dark green, indicating formation of mixed/valent $V^{IV/V}$ species. The solution was filtered and set up for diffusion crystallization using diethyl ether as diffusion solvent. After a few days, black cubic single crystals were obtained. The crystals were recovered by filtration, washed twice with water, ethyl acetate and ether. Upon drying, a grey powder was obtained.

Yield: 530 mg (0.234 mmol, 69 % based on V)

MW: 4499.31g/mol

FT-IR (cm^{-1}): 2960.45, 2933.71, 2871.99, 1481.40, 1460.12, 1379.97, 1150.82, 985.35, 876.58, 818.80, 733.83, 672.65, 602.97, 422.83

Elemental Analysis for $C_{128}H_{289}N_8V_{27}O_{68}Cl_2Mg$ in wt. % (calcd.): C 34.16 (33.97), H 6.43 (6.37), N 2.49 (2.48)

ICP-OES analysis revealed a V : Mg atomic ratio of 27 : 1.17, confirming the expected stoichiometry.

^{1}H-NMR (400 MHz, MeCN-d$_3$): δ 3.20 (8H, nBu$_4$N$^+$), 1.96 (MeCN-d$_3$), 1.65 (8H, nBu$_4$N$^+$), 1.42 (8H, nBu$_4$N$^+$), 1.00 (12H, nBu$_4$N$^+$)

A purified sample of **1** for electrochemistry and EPR analyses was prepared under inert atmosphere in the glove box (to prevent traces of surface-oxidation of the crystals of **1** by air). A sample of **1** was dissolved in water-free, de-aerated acetonitrile. Diffusion of diethyl ether into the solution (in the glove box) gave pure crystals of **1** (verified by single-crystal XRD unit cell checks).

2.1. ¹H-NMR Spectroscopy

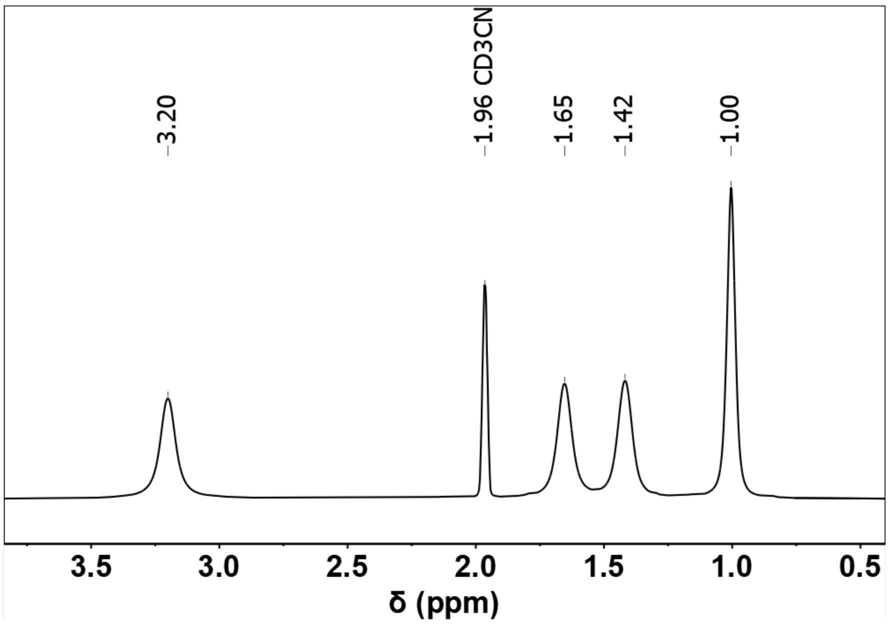

Figure S 1: ¹H NMR of compound **1** in deuterated acetonitrile.

2.2. EPR Spectroscopy

The Easyspin 6.0.2. software suite (https://easyspin.org/) was used to simulate isotropic and fast-motional cw EPR spectra of radicals in solution via the implemented garlic algorithm.[2]

Experimental parameters:
Microwave frequency 9.4463 GHz; microwave power 100 mW; 100kHz modulation amplitude 1 mT.

Simulation parameters for the EPR spectrum of {V_{10}}:

Exchange interactions between two (n = 2) ^{51}V nuclei (I = 7/2; 99.2 % abundance), two hyperfine coupling constants A and two localized electrons indicated by a narrow line width, result in an (2n)I+1 hyperfine coupling pattern.[3] An axial system with two g-values ($g_{\parallel}$ in the direction of the z axis and $g_{\perp}$ in the xy plane were chosen based on the cluster symmetry.[4] Optimum fits were obtained using Lorentzian line shapes with a line width of 34.33 x 10^{-4} cm^{-1}, 103 MHz or 3.83 mT.

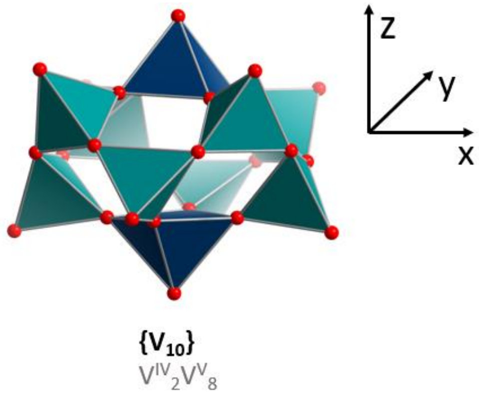

Figure S 2: Structure of the **{V_{10}}** cluster indicating an axial symmetry of the system.[1]

Table S 1: EPR simulation parameters for **$\{V_{10}\}$**.

$g_\perp$	$g_\parallel$	$A_\perp$	$A_\parallel$
1.962	1.950	47.948 x 10^{-4} cm^{-1}<br>144 MHz	42.429 x 10^{-4} cm^{-1}<br>127 MHz

Simulation parameters for the EPR spectrum of compound 1:

For a 1:1 mixture of **$\{MgV_{13}\}$** (3 V^{IV} centers) and **$\{V_{14}\}$** (5 V^{IV} centers) with strong antiferromagnetic coupling and an odd number of electrons for both clusters, a total spin count of 2 x S= ½ (caused by two unpaired electrons) is expected, which is identical to the well-known **$\{V_{10}\}$** reference compound. This was simulated by the combination of two spin systems, both with a weight of 0.5 and S = ½. Due to the interaction of the unpaired electron with the ^{51}V nucleus (I = 7/2; 99.2 % abundance), a hyperfine structure could be expected. However, for **1** no hyperfine structure is observed, indicative of the delocalization of the unpaired electrons over a number of structurally similar V sites. For both spin systems optimum fits were obtained with a gaussian line shape and the following line width lw, isotropic hyperfine interaction A and isotropic g-factor g, see Table S2.

Table S 2: EPR simulation parameters for compound **1**.

Spin system 1			Spin system 2		
g_{iso}	lw	A_{iso}	g_{iso}	lw	A_{iso}
2.025	174.666 x 10^{-4} cm^{-1}<br>524 MHz<br>18.485 mT	36.44 x 10^{-4} cm^{-1}<br>109 MHz	1.890	188.333 x 10^{-4} cm^{-1}<br>565 MHz<br>21.343 mT	30.83 x 10^{-4} cm^{-1}<br>92 MHz

2.3. Thermogravimetric Analysis

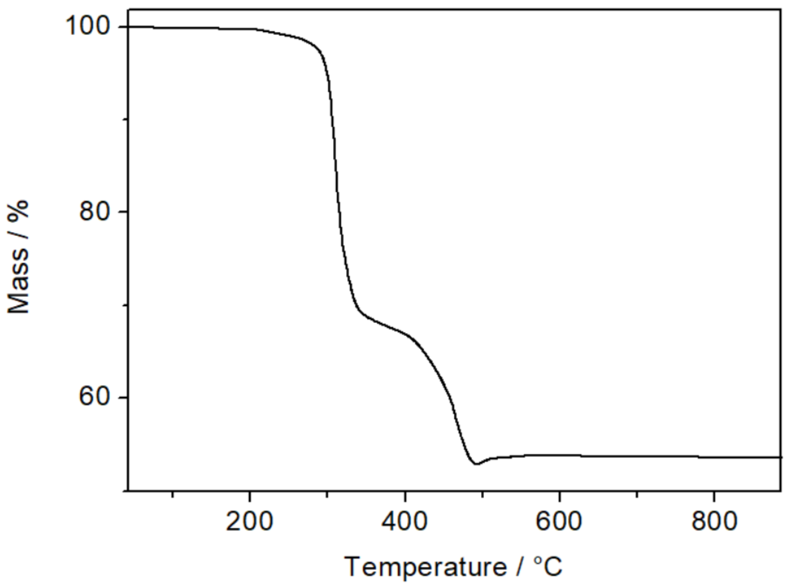

Figure S 3: TGA measurement of compound **1**. The observed weight loss of 45 % between 200 °C and 600 °C indicates the presence of 8 nBu_4N^+ cations in **1** (calcd.: 43 %).

2.4. Cluster Charge Calculations

Based on the EPR results an odd number of V^{IV} centers for each cluster was indicated. The overall amount of eight tetra-n-butylammonium cations to balance the charge results in only three different reasonable electron distributions on the two clusters **{V_{14}}** and **{MgV_{13}}**. Based on the crystallographic analysis (four $n$$Bu_4N^+$ cations are in close proximity of each cluster), a cluster charge of 4- is proposed.

Table S 3: Possible distributions of electrons on **{V_{14}}**.

Possible Formula for **{V_{14}}**	($n$$Bu_4N)_2[V_{14}O_{34}Cl]$	($n$$Bu_4N)_4[V_{14}O_{34}Cl]$	($n$$Bu_4N)_6[V_{14}O_{34}Cl]$
Number of V^V Centers	11	9	7
Number of V^{IV} centers	3	5	7
Total Cluster Charge	2-	4-	6-

Table S 4: Possible distributions of electrons on **{MgV_{13}}**.

Theoretically Calculated Formula of **{MgV_{13}}**	($n$$Bu_4N)_2[(MgOH)V_{13}O_{33}Cl]$	($n$$Bu_4N)_4[(MgOH)V_{13}O_{33}Cl]$	($n$$Bu_4N)_6[(MgOH)V_{13}O_{33}Cl]$
Number of V^V centers	12	10	8
Number of V^{IV} centers	1	3	5
Total Cluster Charge	2-	4-	6-

2.5. ESI Mass Spectrometry

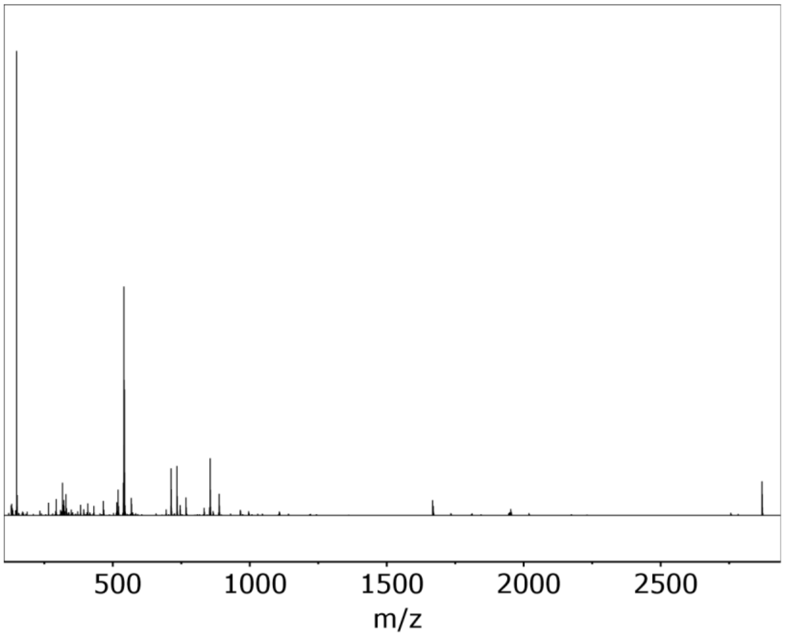

Figure S 4: High-resolution negative-ion mode ESI mass spectrum of **1** (0.05mM) in MeCN.

Table S 5: Detailed peak assignment of the high-resolution negative-ion mode ESI mass spectrum of **1**.

calculated m/z	observed m/z	peak assignment
694.702	694.717	$(^nBu_4N)_3\ [H_2V_7O_{19}]^{2-}$
712.667	712.705	$(^nBu_4N)_3\ [V_7O_{17}Cl_2]^{2-}$
734.203	734.181	$(^nBu_4N)[HV_{13}O_{33}Cl]^{2-}$
745.691	745.671	$(^nBu_4N)[MgV_{13}O_{33}Cl]^{2-}$
753.186	753.158	$\{(^nBu_4N)[MgV_{13}O_{34}Cl]\text{-}H\}^{2-}$
767.170	767.147	$(^nBu_4N)[V_{14}O_{34}Cl]^{2-}$
833.363	833.348	$(^nBu_4N)_2[MgV_{12}O_{32}Cl]^{2-}$
855.346	855.324	$(^nBu_4N)_2[HV_{13}O_{33}Cl]^{2-}$
874.328	874.301	$\{(^nBu_4N)_2[MgV_{13}O_{34}Cl]\text{-}H\}^{2-}$
888.289	888.313	$(^nBu_4N)_2[V_{14}O_{34}Cl]^{2-}$
1667.734	1667.691	$(^nBu_4N)_2[HMgV_{12}O_{32}Cl]^{-}$
1733.668	1733.623	$(^nBu_4N)_2[MgV_{13}O_{34}Cl]^{-}$

Note that the vanadate fragments observed are most likely artefacts formed during the ionization / gas phase transfer process and have been observed for the native **{V$_{12}$}** as well as other related POM species previously.

2.6. UV/Vis/NIR Spectroscopy

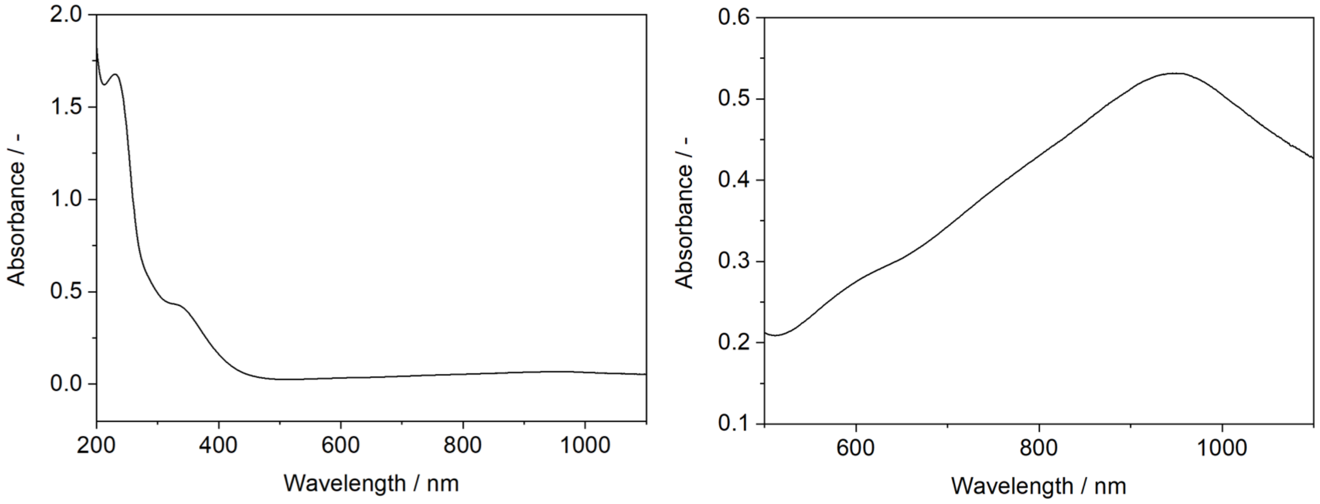

Figure S 5: UV/Vis/NIR spectra of **1** in MeCN. left: [**1**] = 15.625 μmol/L; right: [**1**] = 125 μmol/L.

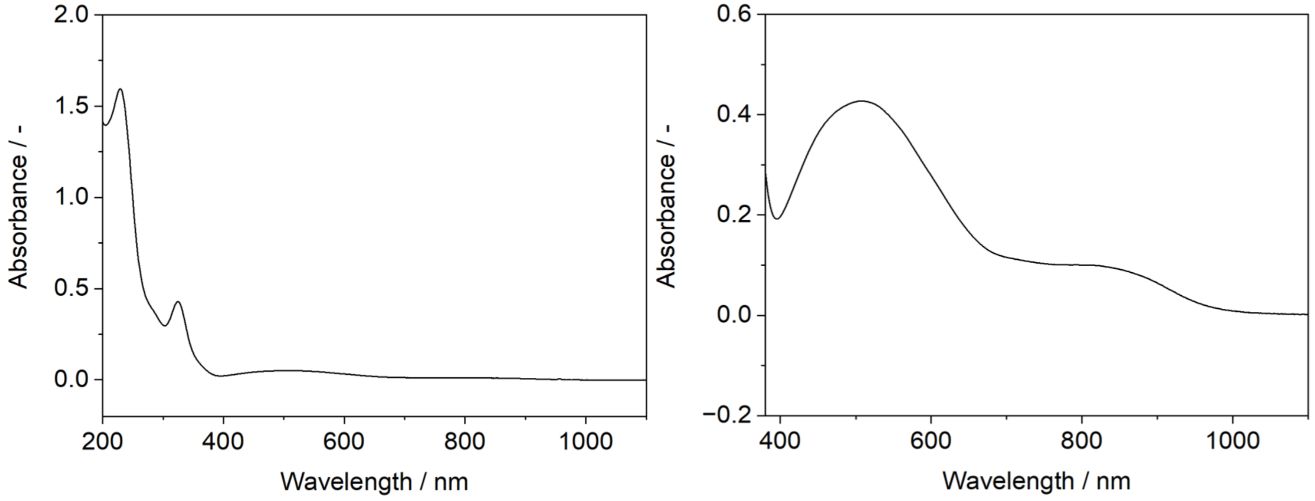

Figure S 6: UV/Vis/NIR spectra of **{V_{10}}** in MeCN. left: [**{V_{10}}**] = 31.25 μmol/L; right: [**{V_{10}}**] = 250 μmol/L.

2.7. Temperature dependent UV/Vis/NIR Spectroscopy

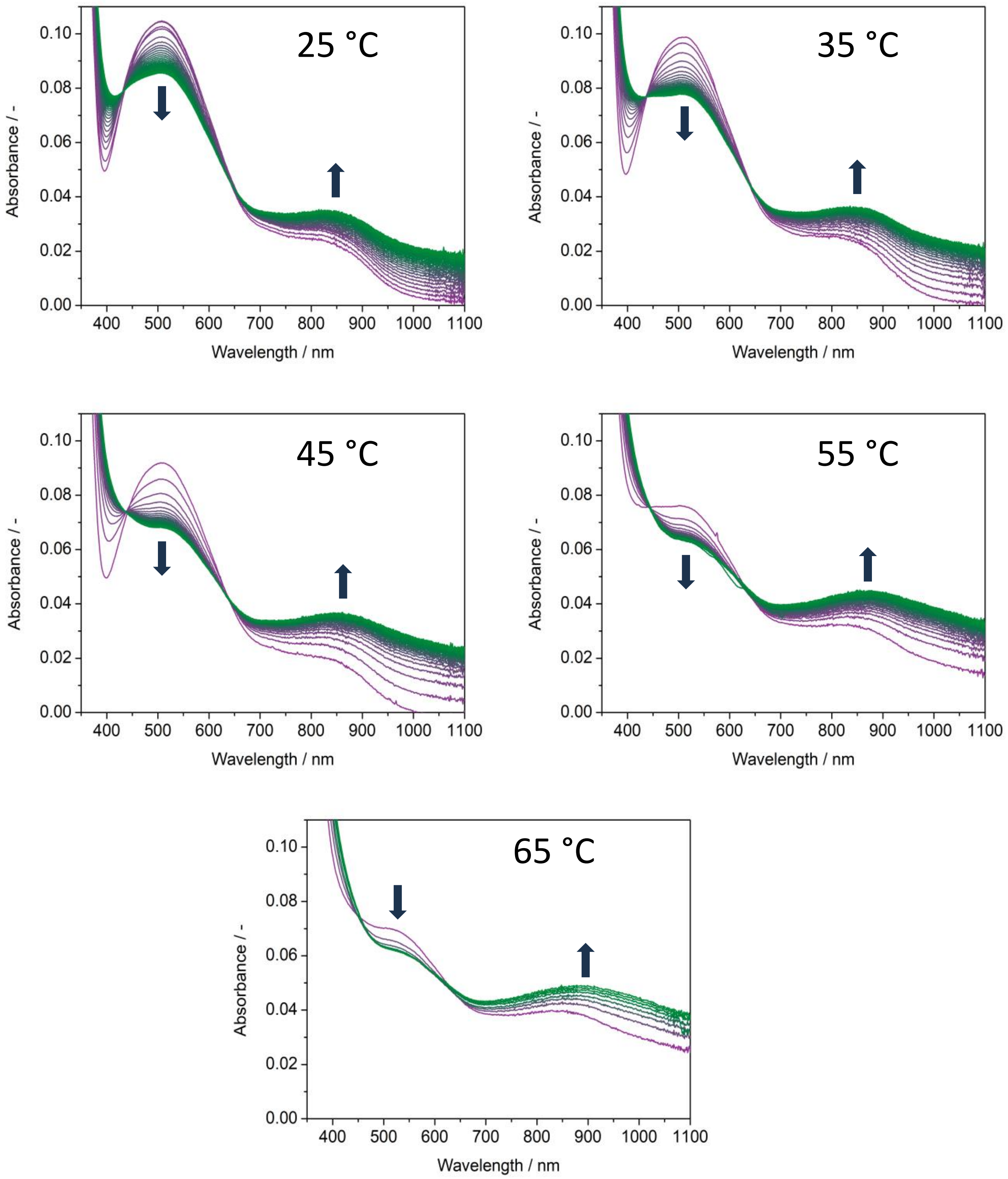

Figure S 7: Temperature dependent UV/Vis-NIR Spectra were measured with a time increment of 60 s, cuvette path length of 2.0 mm, [**{V$_{10}$}**] : [$MgCl_2$ (anhydrous)] = 1 : 2.2 and [**{V$_{10}$}**] = 500 µM.

2.8. Determination of the Molar Activation Energy

Temperature dependent UV/Vis/NIR experiments were evaluated using the Arrhenius Equation.[5]

Arrhenius equation:

Rate constant = k
Absolute Temperature = T
Pre-exponential factor = k_0
Molar activation energy = E_a
Universal gas constant = R

$$k = k_0 e^{-\frac{E_a}{RT}}$$

Exponential decay fit function for a reaction of first order assuming the decomposition of **{V10}** in presence of Mg^{2+} as the rate determining step:

$$y = y_0 + k_0 e^{-kt}$$

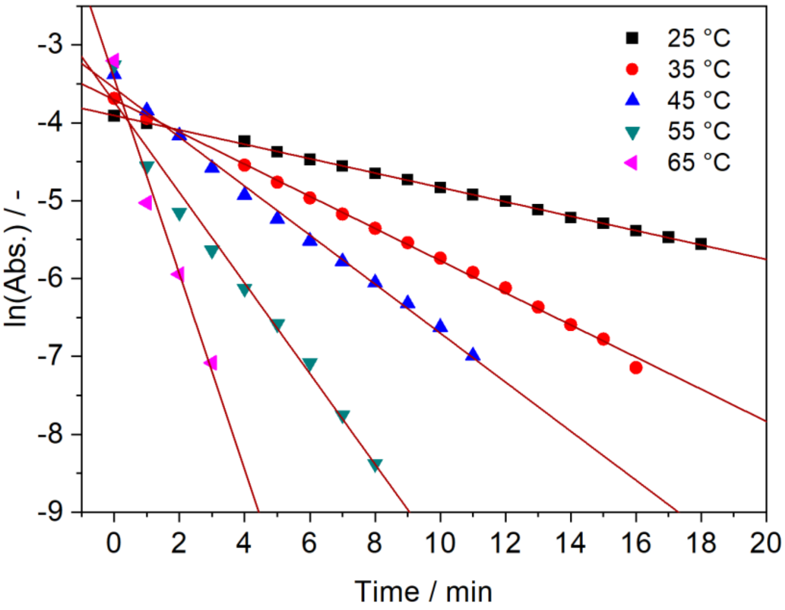

Figure S 8: Linear change of ln(Abs (λ = 500 nm)) *vs* time confirms the first order kinetics.

Table S 6: The fit parameters of the mono-exponential fit **Figure 4d** were used for the Arrhenius Plot **Figure 4c**.

**Temperature dependent Exponential Decay Fit Parameters**						
Temperature / °C	y_0	Δy_0	k_0	Δk_0	k	Δk
25	0.08419	3.10467E-4	0.02007	2.75406E-4	0.09191	0.00304
35	0.07918	1.31221E-4	0.02458	2.08578E-4	0.20856	0.00438
45	0.07022	2.12505E-4	0.03309	5.84747E-4	0.37575	0.01379
55	0.06567	2.83053E-4	0.0381	0.00115	1.10502	0.08029
65	0.06353	2.94462E-4	0.04062	7.84384E-4	1.74749	0.10992

Table S 7: The fit parameters of the Arrhenius Plot were used to calculate the activation energy.

Linear Fit Parameters of the Arrhenius Plot and Calculated Molar Activation Energy					
$-\frac{E_a}{R}$ / 1/K	$\Delta -\frac{E_a}{R}$ / 1/K	ln(A) / -	Δ ln(A) / -	E_a / kJ/mol	ΔE_a / kJ/mol
-7620.4453	-467.37833	23.1448	1.47342	63.356	3.886

2.9. Mg^{2+} Concentration-dependent UV/Vis/NIR

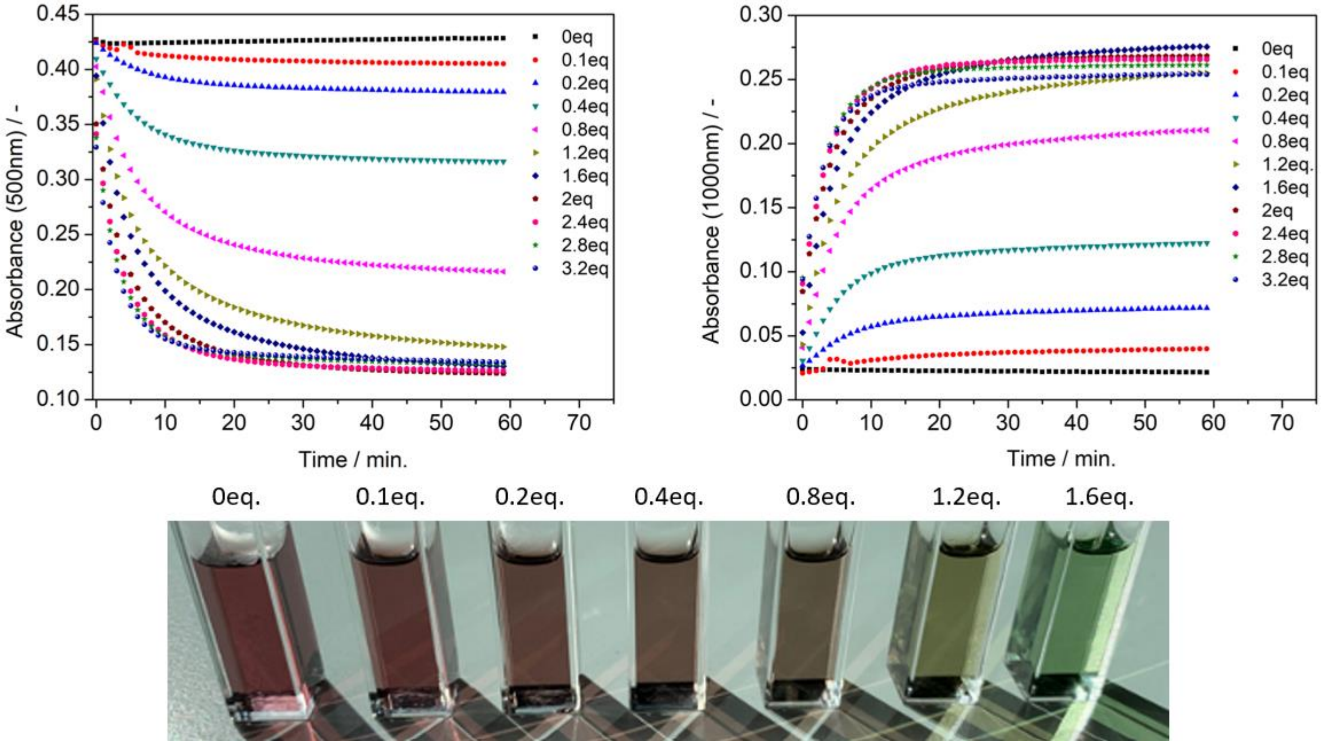

Figure S 9: UV/Vis/NIR of **{V_{10}}** was measured during the equilibration time of 1h with a time interval of 1 min at [**{V_{10}}**] = 230 µM, a temperature of 35 °C and a cuvette pathlength of 10 mm pathlength.

2.10. ATR-FT-IR spectroscopy

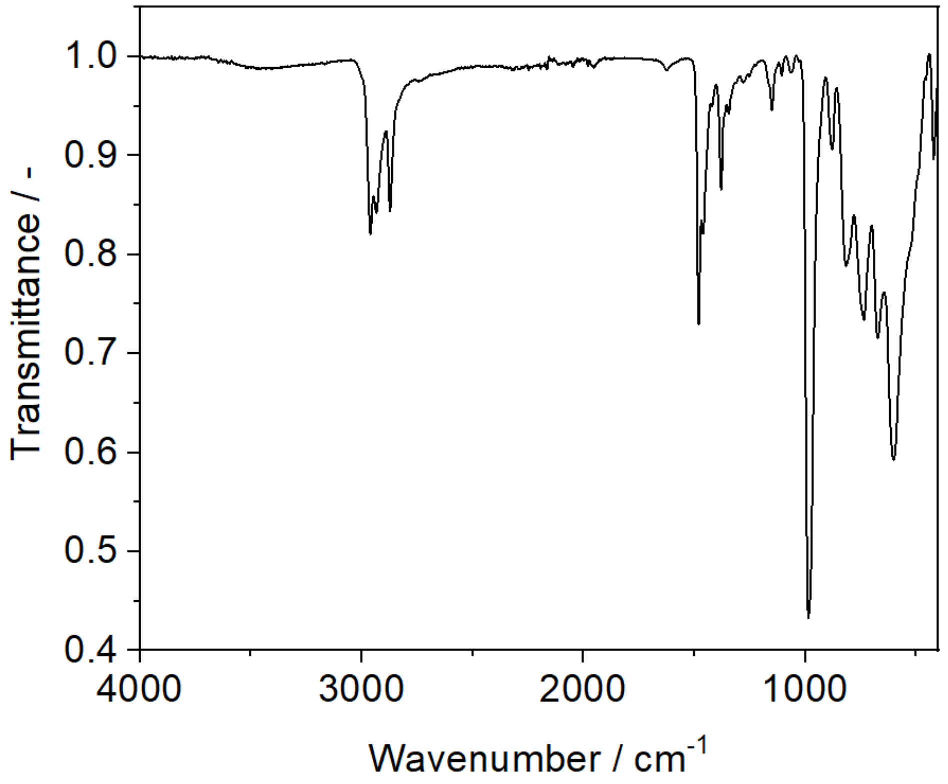

Figure S 10: ATR-FT-IR spectrum of compound **1**.

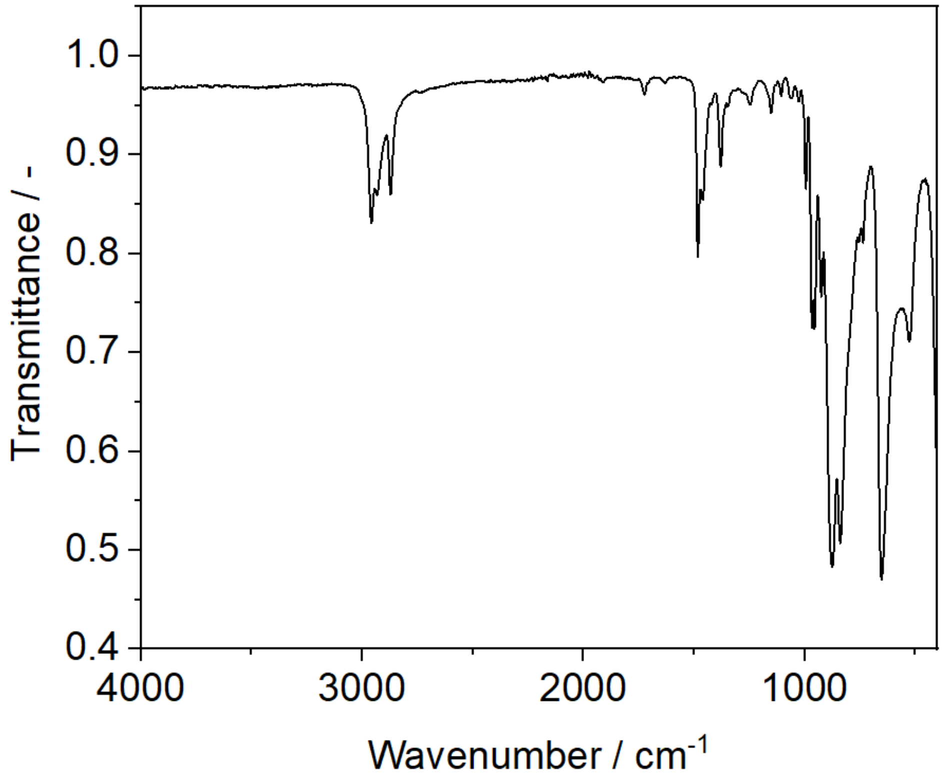

Figure S 11: ATR-FT-IR spectrum of **{V_{10}}**.

2.11. Electrochemistry

Electrochemical intervals in one-component POMs

Table S 8: Summary of electrochemical intervals between individual redox events in selected classical single-cluster polyoxometalates.

Entry	Abbreviation of POM	Average $\Delta E_{1/2}$ [V]	Reference
1	α-$[PW_{12}O_{40}]^{3-}$	0.55	T. Ueda et.al [6]
2	$H_3[PMo_{12}O_{40}]$	0.30	M. A. Barteau et.al [7]
3	α-$[W_{18}O_{54}(SO_3)_2]^{5-}$	0.45	L. Cronin et.al [8]
4	$[Ca_2V_{12}O_{32}Cl(DMF)_3]^{2-}$	0.41	C. Streb et.al [9]
5	$[V_6O_7(OEt)_{12}]$	0.56	E. Matson et.al [10]
6	$(NEt_4)_5[V_{18}O_{42}(I)]$	0.37	K.Y. Monakhov et.al [11]
7	$(n\text{Bu}_4N)_8[(MgOH)V_{13}O_{33}Cl][V_{14}O_{34}Cl]$ (compound **1**)	0.22	This work

Voltammetry

CV and SWV of **1** show fourteen quasi-reversible redox transitions in the potential range from -2.15 V to + 1.35 V (0.1 M *n*Bu_4PF_6 in MeCN): $E_{1/2}$ (vs. Fc/Fc$^+$) = - 1.98 V; - 1.76 V; - 1.55 V; - 1.33 V; - 1.08 V; - 0.83 V; - 0.74 V; - 0.51 V; - 0.30 V; - 0.20 V; + 0.18 V; + 0.32 V; + 0.62 V; + 0.82 V.

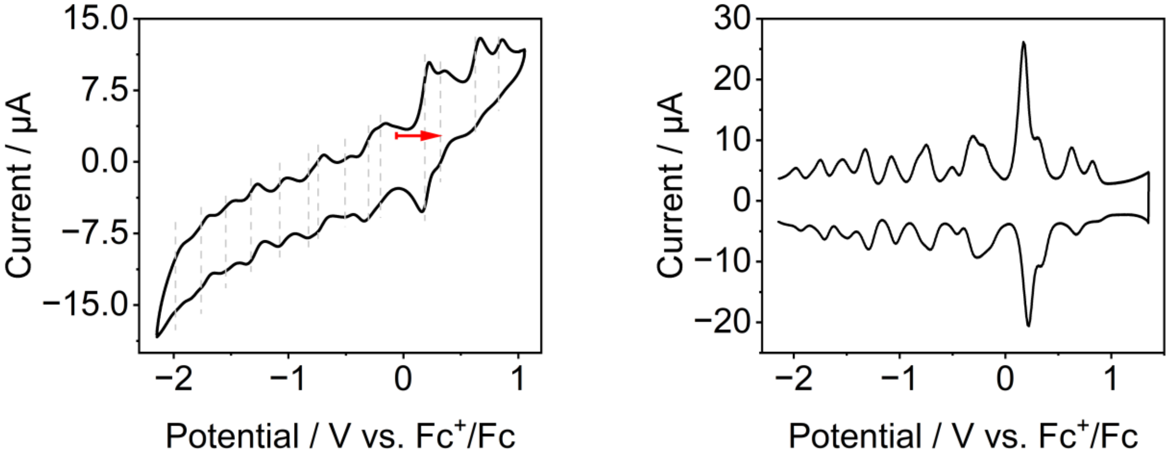

Figure S 11: Cyclic voltammogram (left) and square wave voltammogram (right) of **1**. Conditions: anhydrous, deoxygenated acetonitrile containing nBu_4NPF_6 (0.1 M) as supporting electrolyte (scan rate 0.05 V s^{-1}), [**1**]: 0.5 mM.

2.12. Bulk Electrolysis

Bulk Reduction (BR) of 1

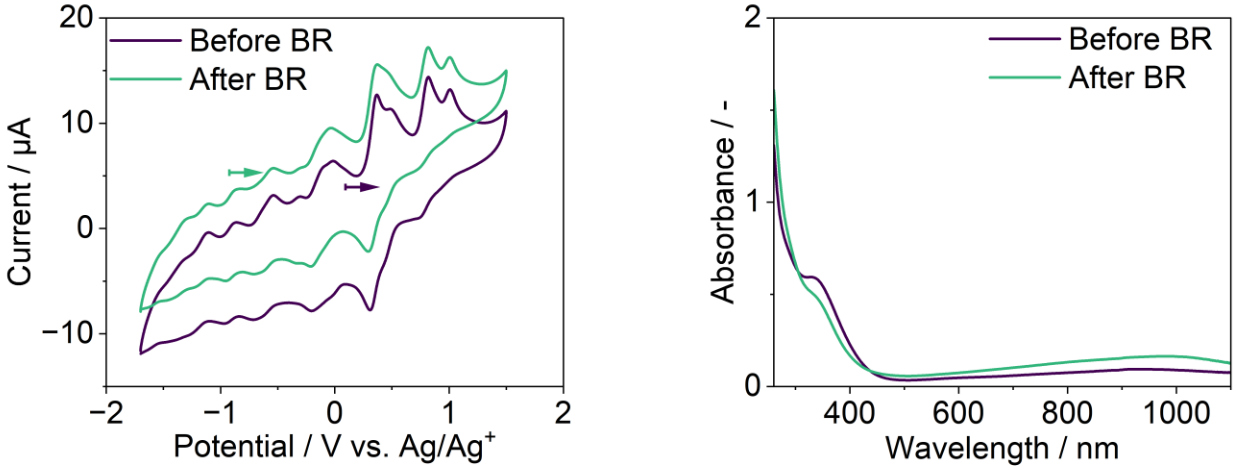

Figure S 12: Left: Comparison of CVs of **1** before (dark blue) and after (turquoise) bulk reduction (BR) (E = - 1.45 V). Open circuit potentials and scan directions are indicated by arrows. Right: UV/Vis/NIR absorption spectra of **1** before (dark blue) and after (turquoise) bulk reduction.

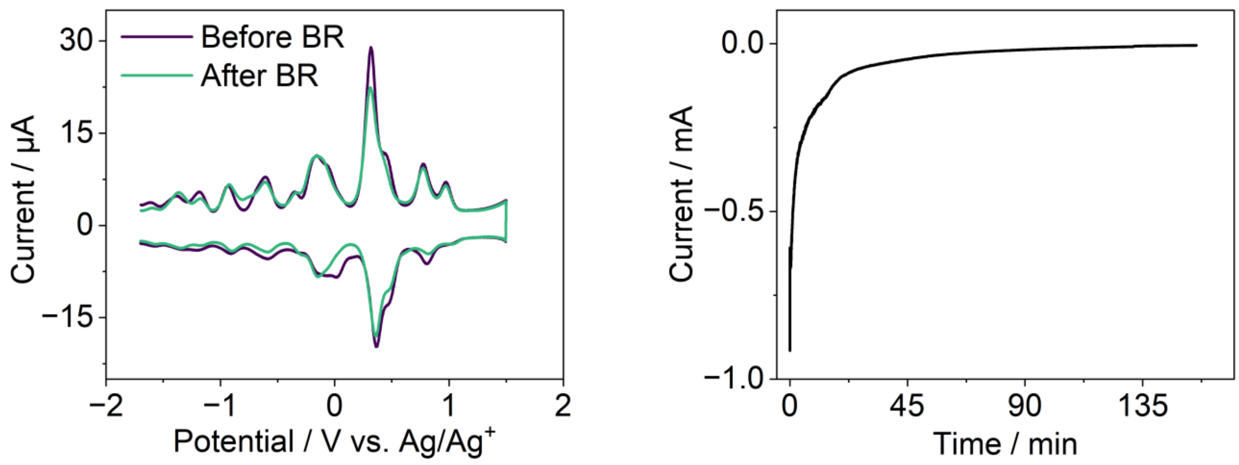

Figure S 13: Left: Square wave voltammogram of **1** before (dark blue) and after (turquoise) bulk reduction (E = - 1.45 V). Right: Chronoamperogram of bulk electrolysis (E = -1.45 V).

Bulk Oxidation (BOx) of 1

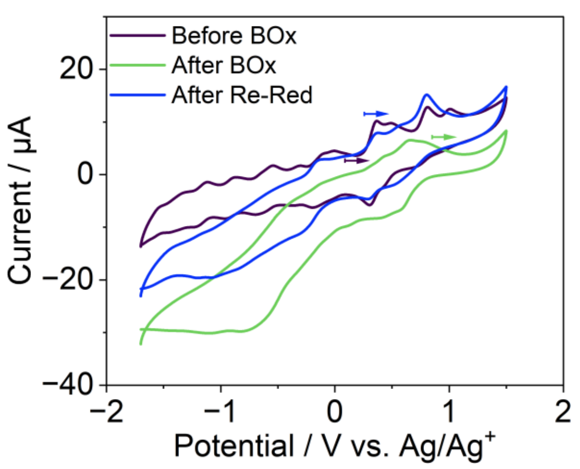

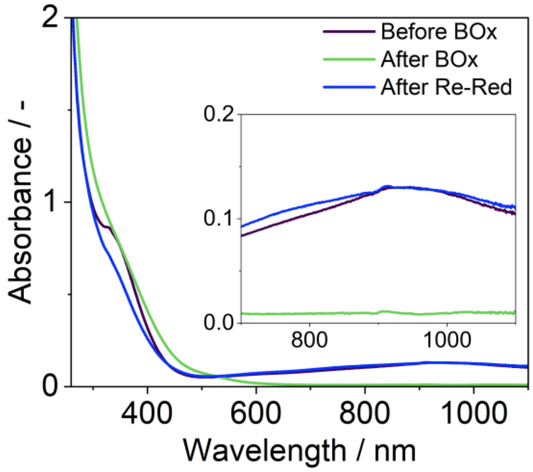

Figure S 14: Left: Comparison of CVs of **1** before (dark blue) and after (green) bulk oxidation (BOx) (*E* = + 1.22 V) and after re-reduction (Re-Red) (blue) (*E* = + 0.09 V). Open circuit potentials and scan directions are indicated by arrows. Right: UV/Vis/NIR absorption spectra of **1** before (dark blue) and after (green) bulk oxidation.

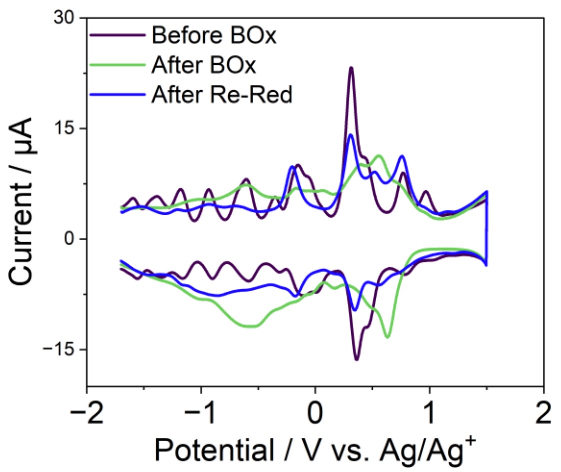

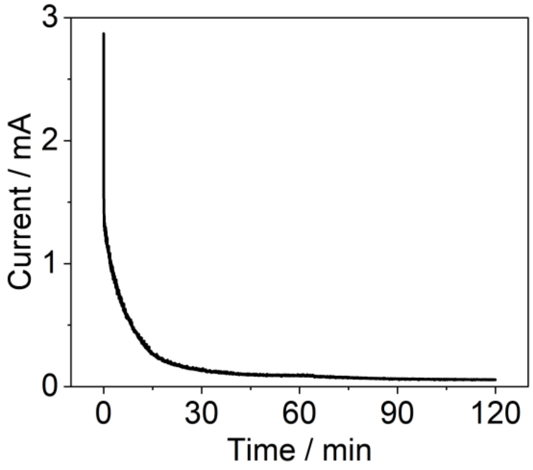

Figure S 15: Left: Square wave voltammogram of **1** before (dark blue) and after (green) bulk electrolysis (*E* = + 1.22 V) and after re-reduction (Re-Red) (blue) (*E* = + 0.09 V). Right: Chronoamperogram of bulk electrolysis (*E* = + 1.22 V).

Table S 9: Summary of bulk electrolyses with calculated average number of transferred electrons per cluster.

Entry	Type of Electrolysis	Average # of transferred e^- / cluster	Average # of transferred e^- / formula unit of 1 (containing one **{MgV_{13}}** and one **{V_{14}}** cluster)
1	Bulk Reduction (*E* = - 1.45 V)	3.93 ± 0.42	7.86 ± 0.84
2	Bulk Oxidation (*E* = +1.22 V)	4.33 ± 0.43[a]	8.66 ± 0.86[a]

[a] the slightly increased number of electrons transferred during bulk oxidation is currently assigned to undesired oxidative side reactions.

Integration of square wave voltammetry data

To gain initial insights into the number of electrons transferred during the redox transitions in compound 1, the oxidative branch of the square wave voltammogram (Figure S11) was integrated. In Figure S16, colored potential ranges indicate each integration region. The integrals cover all 14 observed redox transitions.

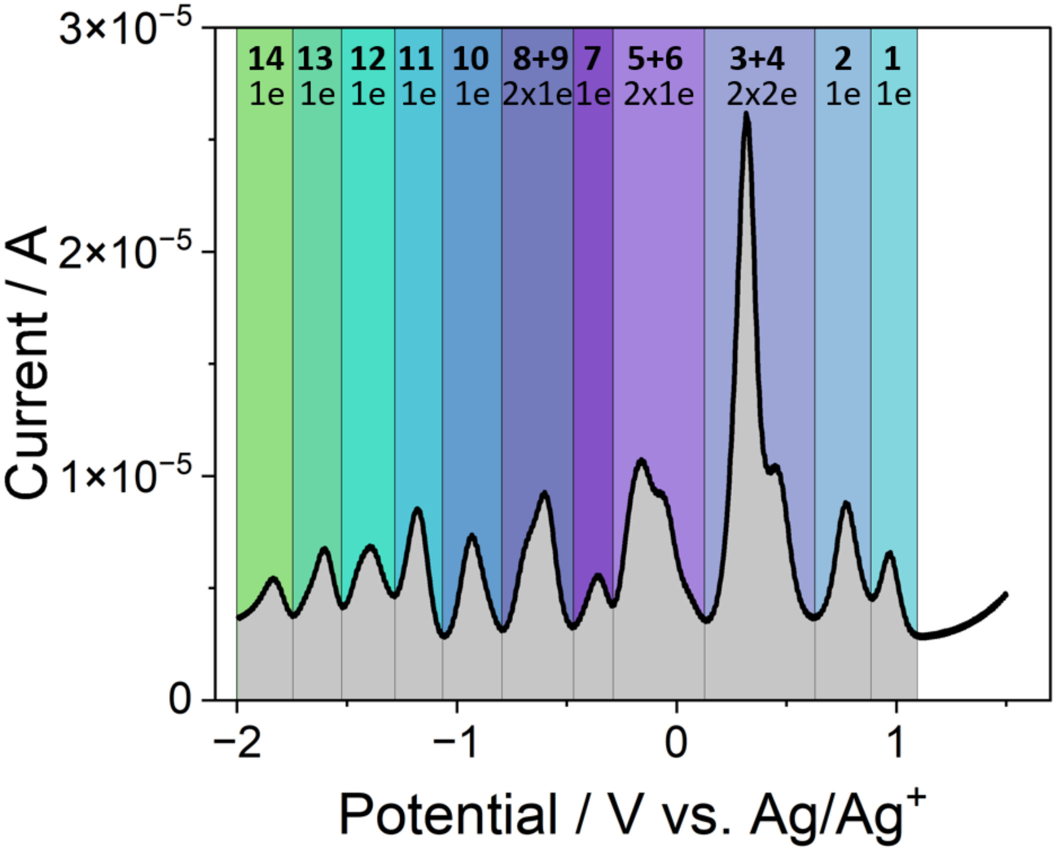

Figure S 16: Upper segment of square-wave voltammogram of **1** showing the integration limits indicated by differently colored regions. The integrals were used to estimate the numbers of electrons transferred for each process, see below.

To estimate the number of electrons transferred in each observed redox transition, we first determined an average integral area assigned for an expected 1-electron transfer. This mean integral area A_{mean} was obtained as average of well-separated signals 1,2,7, 10, 11, 12, 13.
This analysis gave A_{mean} [A·V] = 1.20 x 10^{-6} ± 0.23 x 10^{-6}
This value was used to normalize each integral area observed for the individual redox transitions as shown in Table S 10. Note that processes 3 and 4, 5 and 6, 8 and 9 could not be separated as they partially overlap, as shown in Figure S16. Electron transfer numbers were then rounded to the nearest integer number to give the nominal number of electrons transferred. Note that integration of the SWV data is challenging due to the complex nature of the voltammogram, the close spacing and partial overlap of the signals observed.

Table S 10: Summary of the integration of the square wave voltammogram of **1**, and the resulting electron transfer numbers for **1**.

**Redox Event**	**Integral range**		**Integral Area [A·V]**	**Estimated number of electrons transferred[a]**	**Nominal number of electrons transferred[b]**
	***E*₁ [V]**	***E*₂ [V]**			
1	0.885	1.090	9.85E-07	0.82	1
2	0.630	0.885	1.54E-06	1.28	1
3+4	0.128	0.630	5.17E-06	2 x 2.15	2 x 2
5+6	-0.289	0.128	3.03E-06	2 x 1.26	2 x 1
7	-0.469	-0.289	8.24E-07	0.69	1
8+9	-0.794	-0.469	1.95E-06	2 x 0.82	2 x 1
10	-1.065	-0.794	1.32E-06	1.10	1
11	-1.281	-1.065	1.29E-06	1.08	1
12	-1.524	-1.281	1.36E-06	1.13	1
13	-1.747	-1.524	1.16E-06	0.97	1
14	-2.000	-1.747	1.11E-06	0.93	1
			**Sum**	**16.47**	**16**

[a] calculated as Integral Area divided by A_{mean}
[b] Obtained by rounding the estimated number of electrons transferred to the nearest integer number.

2.13. Crystallographic Information

Suitable single crystals were mounted onto a microloop using Fomblin oil. X-ray diffraction intensity data were measured at 150 K on a Bruker D8 QUEST diffractometer λ(MoKα = 0.71073 Å) equipped with a graphite monochromator. Structure solution was carried out using SHELX-2013[12] package through OLEX2.[13,14] Corrections for incident and diffracted beam absorption effects were applied using empirical methods.[15] Structures were solved by a combination of direct methods and difference Fourier syntheses and refined against F^2 by the full matrix least-squares technique. Most non-hydrogen atoms were refined anisotropically. A two-component twin model was used for the refinement. The metal oxo framework was refined fully anisotropically. Restraints (SIMU and DELU) on the counter cations were applied. The CIF files can be obtained free of charge from the CCDC, reference number CCDC 2369209.

Table S10: Crystallographic parameters for the samples

Empirical formula	$C_{126}Cl_2Mg_{0.84}N_8O_{68}V_{27.2}$
CCDC reference no	2369209
Formula weight	4188.27
Temperature/K	150.00
Crystal system	monoclinic
Space group	*C*2
a/Å	23.189(4)
b/Å	23.197(4)
c/Å	17.660(3)
α/°	90
β/°	90.089(6)
γ/°	90
Volume/Å^3	9500(3)
Z	4
T / K	150(1)
ρ_{calc}g/cm^3	1.464
μ/mm^{-1}	1.370
F(000)	4668.0
Radiation	MoKα (λ = 0.71073)
2Θ range for data collection/°	4.6 to 52.8
Index ranges	-28 ≤ h ≤ 28, -29 ≤ k ≤ 29, -22 ≤ l ≤ 22
Reflections collected	94631
Independent reflections	19413 [R_{int} = 0.0980, R_{sigma} = 0.0704]
Data/restraints/parameters	15471/905/1069
Goodness-of-fit on F^2	1.093
Final R indexes [I>=2σ (I)]	R_1 = 0.0822, wR_2 = 0.2142
Final R indexes [all data]	R_1 = 0.1084, wR_2 = 0.2492
Largest diff. peak/hole / e Å^{-3}	1.24/-0.85

2.14. Electrochemical reference studies

To assess the effects of simple physical mixing of two structurally or chemically related polyoxovanadate clusters, the following studies were performed. 1:1 molar mixtures of the related species $[H_3V_{10}O_{28}]^{3-}$[16] and **{V₁₀}**, as well as **$\{V_{12}\}$**$_{half\text{-}sphere}$[17] and **$\{Ca_2V_{12}\}$**[18] were prepared and analyzed by SWV, see Figure S17. The data show that part of the electrochemical signals is retained, while ^{51}V NMR analysis of the reaction solutions indicates that partial degradation and/or conversion of the original clusters is observed, thus the solutions are not fully redox-stable.

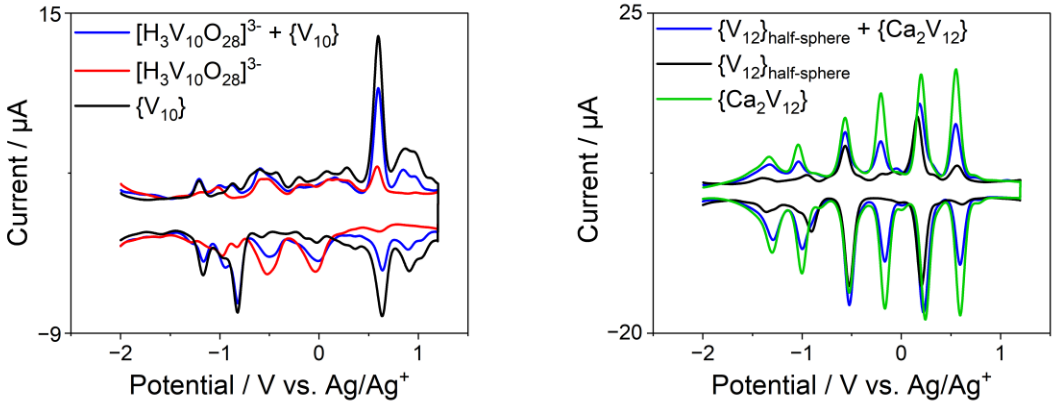

Figure S 17: **Left:** Square wave voltammograms of $[H_3V_{10}O_{28}]^{3-}$, **$\{V_{10}\}$** and their 1:1 molar mixture. **Right:** Square wave voltammograms of **$\{V_{12}\}_{half\text{-}sphere}$**, **$\{Ca_2V_{12}\}$** and their 1:1 molar mixture. Solvent: De-aerated, degassed and dry MeCN containing $(nBu_4N)PF_6$ (0.1 M).

2.15. Computational Section

Geometry optimizations of the clusters in the gas-phase and solution phase were carried out using density functional theory calculations with the B3LYP[19] functional combined with the def2-SVP basis set.[20] Solvation effects were considered using the SMD implicit solvation model (acetonitrile).[21] All calculations were performed using the Gaussian 16 package. [22]

Computational redox potentials: One-electron redox process of a redox reaction can be simply defined in terms of a half-cell reaction as follows:

$\mathrm{Oxd}^{m} + e^- \rightarrow \mathrm{Red}^{m-1}$

Where Oxd is an oxidized species and Red is a reduced species. The absolute redox potential $E_{\mathrm{ET}}^{\mathrm{abs}}$ of this redox couple is calculated by eq. (1), where $\Delta G^0_{(s)}$ is the free energy change in solution and F is Faraday's constant:

$$E_{\mathrm{ET}}^{\mathrm{abs}} = -\frac{\Delta G^{0}(s)}{F} \quad (1)$$

The thermodynamic cycle shown below is used to calculate reduction potentials. This is a schematic illustration of gas-phase and solution-phase reactions, as well as the relation between the phases.

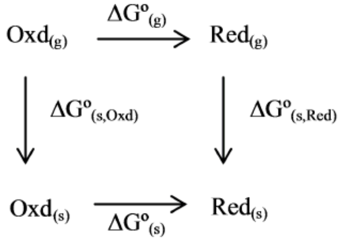

Figure S18: Thermodynamic cycle for the calculation of Gibbs free energies of a one-electron reduction process.

Based on the thermodynamic cycle, $\Delta G^0_{(s)}$ can be expressed as

$$\Delta G^0_{(s)} = \Delta G^0_{(g)} + \Delta G^0_{(s,\ Red)} - \Delta G^0_{(s,\ Oxd)} \qquad (2)$$

Where $\Delta G^0_{(s)}$ is the free energy change in the gas phase, while $\Delta G^0_{(s,Oxd)}$ and $\Delta G^0_{(s,Red)}$ are the solvation free energies of the oxidized and reduced species in acetonitrile, respectively.

Experimentally, the redox potentials are quoted relative to the Fc^+/Fc external standard with a reference potential of $E^{abs}_{exp,RE}$ = 0.40 V relative to the standard hydrogen electrode.[23] The theoretical redox potentials with respect to the experimental reference are calculated using equation (3):

$$E^{calc}_{ET}(\mathrm{V}\ vs.\ \mathrm{Fc/Fc^+}) = E^{abs}_{ET} - E^{abs}_{SHE} - E^{abs}_{exp,RE} \qquad (3)$$

Where E^{abs}_{SHE} denotes the absolute electrode potential of the standard hydrogen electrode.

The experimental and calculated reduction potential of $\{V_{14}\}$ and $\{MgV_{13}\}$ are summarized in table S11.

Table S11. Experimental and calculated reduction potentials for **$\{V_{14}\}$** and **$\{MgV_{13}\}$**.

No	Assignment	$E_{exp.}$ / V[a]	$E_{calcd.}$ / V[a]
**8**	$\{MgV_{13}\}^{4-} + e^- \rightarrow \{MgV_{13}\}^{5-}$	−0.73	−0.68
**9**	$\{V_{14}\}^{4-} + e^- \rightarrow \{V_{14}\}^{5-}$	-0.83	-0.81

[a] E_{red} *vs.* Fc^+/Fc.

3. References

[1] K. Okaya, T. Kobayashi, Y. Koyama, Y. Hayashi, K. Isobe, *Eur J Inorg Chem* **2009**, *2009*, 5156–5163.
[2] S. Stoll, A. Schweiger, *Journal of Magnetic Resonance* **2006**, *178*, 42–55.
[3] R. L. Belford, N. D. Chasteen, H. So, R. E. Tapscott, *J Am Chem Soc* **1969**, *91*, 4675–4680.
[4] Q. Chen, D. P. Goshorn, C. P. Scholes, X. L. Tan, J. Zubieta, *J Am Chem Soc* **1992**, *114*, 4667–4681.
[5] K. J. Laidler, *J Chem Educ* **1984**, *61*, 494.
[6] T. Ueda, K. Kodani, H. Ota, M. Shiro, S.-X. Guo, J. F. Boas, A. M. Bond, *Inorg Chem* **2017**, *56*, 3990–4001.
[7] Y. Cao, J.-J. J. Chen, M. A. Barteau, *Journal of Energy Chemistry* **2020**, *50*, 115–124.

[8] N. Fay, A. M. Bond, C. Baffert, J. F. Boas, J. R. Pilbrow, D.-L. Long, L. Cronin, *Inorg Chem* **2007**, *46*, 3502–3510.
[9] S. Greiner, B. Schwarz, M. Ringenberg, M. Dürr, I. Ivanovic-Burmazovic, M. Fichtner, M. Anjass, C. Streb, *Chem Sci* **2020**, *11*, 4450–4455.
[10] L. E. VanGelder, A. M. Kosswattaarachchi, P. L. Forrestel, T. R. Cook, E. M. Matson, *Chem Sci* **2018**, *9*, 1692–1699.
[11] O. Linnenberg, M. Moors, A. Solé-Daura, X. López, C. Bäumer, E. Kentzinger, W. Pyckhout-Hintzen, K. Yu. Monakhov, *The Journal of Physical Chemistry C* **2017**, *121*, 10419–10429.
[12] G. M. Sheldrick, *Acta Crystallogr A* **2008**, *64*, 112–122.
[13] O. V. Dolomanov, L. J. Bourhis, R. J. Gildea, J. A. K. Howard, H. Puschmann, *J Appl Crystallogr* **2009**, *42*, 339–341.
[14] L. J. Bourhis, O. V. Dolomanov, R. J. Gildea, J. A. K. Howard, H. Puschmann, *Acta Crystallogr A Found Adv* **2015**, *71*, 59–75.
[15] R. H. Blessing, *Acta Crystallogr A* **1995**, *51*, 33–38.
[16] V. W. Day, W. G. Klemperer, D. J. Maltbie, *J Am Chem Soc* **1987**, *109*, 2991–3002.
[17] V. W. Day, W. G. Klemperer, O. M. Yaghi, *J Am Chem Soc* **1989**, *111*, 5959–5961.
[18] S. Greiner, B. Schwarz, M. Ringenberg, M. Dürr, I. Ivanovic-Burmazovic, M. Fichtner, M. Anjass, C. Streb, *Chem Sci* **2020**, *11*, 4450–4455.
[19] A. D. Becke, *J. Chem. Phys.* **1993**, *98*, 5648–5652.
[20] F. Weigend, R. Ahlrichs, *Physical Chemistry Chemical Physics* **2005**, *7*, 3297.
[21] A. V. Marenich, C. J. Cramer, D. G. Truhlar, *Journal of Physical Chemistry B* **2009**, *113*, 6378–6396.
[22] M. J. Frisch, G. W. Trucks, H. B. Schlegel, V. B. M. A. Robb, J. R. Cheeseman, G. Scalmani, A. V. M. G. A. Petersson, H. Nakatsuji, X. Li, M. Caricato, H. P. H. J. Bloino, B. G. Janesko, R. Gomperts, B. Mennucci, D. W.-Y. J. V. Ortiz, A. F. Izmaylov, J. L. Sonnenberg, A. P. F. Ding, F. Lipparini, F. Egidi, J. Goings, B. Peng, N. R. T. Henderson, D. Ranasinghe, V. G. Zakrzewski, J. Gao, R. F. G. Zheng, W. Liang, M. Hada, M. Ehara, K. Toyota, H. N. J. Hasegawa, M. Ishida, T. Nakajima, Y. Honda, O. Kitao, J. E. P. T. Vreven, K. Throssell, J. A. Montgomery, Jr., K. N. K. F. Ogliaro, M. J. Bearpark, J. J. Heyd, E. N. Brothers, J. N. V. N. Staroverov, T. A. Keith, R. Kobayashi, S. S. I. K. Raghavachari, A. P. Rendell, J. C. Burant, R. C. J. Tomasi, M. Cossi, J. M. Millam, M. Klene, C. Adamo, O. F. J. W. Ochterski, R. L. Martin, K. Morokuma, and D. J. F. J. B. Foresman, *Gaussian 16, Revision A.03, Gaussian, Inc., Wallingford CT* **2016**.
[23] R. M. Olson, A V Marenich, C. J. Cramer, D. G. Truhlar, *J Chem Theory Comput* **2007**, *3*, 2046–2054.
